# The demography of human warfare can drive sex differences in altruism

**DOI:** 10.1017/ehs.2020.5

**Published:** 2020-02-20

**Authors:** Alberto J. C. Micheletti, Graeme D. Ruxton, Andy Gardner

**Affiliations:** 1 School of Biology, University of St Andrews, Dyers Brae, St Andrews KY16 9TH, UK; 2 Institute for Advanced Study in Toulouse, Université Toulouse 1 Capitole, 1 esplanade de l'Université, 31080 Toulouse Cedex 06, France

**Keywords:** War, cooperation, parochial altruism, sex-biased dispersal, kin competition

## Abstract

Recent years have seen great interest in the suggestion that between-group aggression and within-group altruism have coevolved. However, these efforts have neglected the possibility that warfare – via its impact on demography – might influence human social behaviours more widely, not just those directly connected to success in war. Moreover, the potential for sex differences in the demography of warfare to translate into sex differences in social behaviour more generally has remained unexplored. Here, we develop a kin-selection model of altruism performed by men and women for the benefit of their groupmates in a population experiencing intergroup conflict. We find that warfare can promote altruistic, helping behaviours as the additional reproductive opportunities winners obtain in defeated groups decrease harmful competition between kin. Furthermore, we find that sex can be a crucial modulator of altruism, with there being a tendency for the sex that competes more intensely with relatives to behave more altruistically and for the sex that competes more intensely with non-relatives in defeated groups to receive more altruism. In addition, there is also a tendency for the less-dispersing sex to both give and receive more altruism. We discuss implications for our understanding of observed sex differences in cooperation in human societies.

## Graphical abstract

**Figure:**
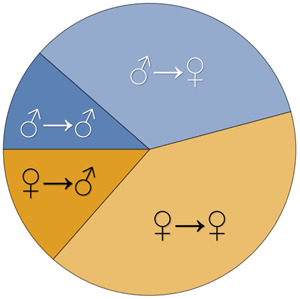

**Media summary:** Recent research has suggested that altruism and warfare might have evolved together. However, by focusing only on behaviours that improve success in battle, this work has ignored the potential for warfare to influence altruism more widely – and has also neglected the possible role of sex differences in this context. We developed a mathematical evolutionary model to explore these questions. We found that men, the sex that historically participated in war and also migrated less often, would have tended to be more altruistic and also receive more help from other men – forming ‘boys’ clubs’. Depending on the nature of warfare and migration, other systems are also possible, including societies where it is mainly men who help women or even ‘girls’ clubs’.

## Introduction

The last decade has seen considerable multidisciplinary interest in understanding the potential evolutionary links between warfare and the high levels of altruism observed in human societies (Bauer *et al*. 2016; Rusch 2014; Rusch *et al.* 2016; Turchin 2015). Darwin (1871) was the first to explore this possibility, suggesting that intergroup conflict might have generated selection for altruism, on account of more cooperative groups being more successful in warfare. Recently, this idea has been modelled mathematically by Choi and Bowles (2007), who showed that within-group altruism and between-group hostility could have coevolved, leading to an intersection of these two behaviours that has been termed ‘parochial altruism’ (see also Bowles 2006, 2009; Garcia and van der Bergh 2011). Empirical evidence for the co-occurrence of these behaviours in both small-scale and industrialised societies is mixed and still subject to investigation (Doğan *et al.* 2018; Gneezy and Fessler, 2011; Mathew and Boyd 2011; Romano *et al.* 2017; Schaub 2017; Silva and Mace 2014; also reviewed in Rusch 2014; Rusch *et al.* 2016).

However, the possibility that warfare, through its effect on demography, might shape human social behaviours more widely than just those connected with success in war has been largely neglected. Warfare can affect with whom individuals mate and whether or not they reproduce at all (Glowacki *et al.* 2017), thus possibly altering patterns of relatedness and competition. Patterns of relatedness and competition, in turn, are known to modulate incentives to perform social behaviours (Frank 1998) and, in this way, warfare demographies could influence other forms of within-group altruism – not just those that result in greater success in battle and that have been the focus of mathematical analyses so far (also Bowles 2006, 2009; Choi and Bowles 2007; Garcia and van der Bergh 2011; Lehmann and Feldman 2008; Micheletti *et al.* 2017, 2018). Moreover, incentives to behave altruistically could vary in sex-specific ways, as warfare might affect the demography of the two sexes differently – for example through sex-biased dispersal. While sex has been suggested to modulate behaviours in the context of intergroup conflict in both empirical (Johnson *et al.* 2006; van Vugt *et al.*^2006^; McDonald *et al.* 2012; van Vugt 2009) and theoretical studies (Micheletti *et al.* 2017, 2018), the role of sex-biased demographies in this respect has not been explored formally. Furthermore, the intensity of altruistic behaviours might depend on the sex of the recipient of altruism, as well as on the sex of the altruist – that is, the sex of both interactants may determine behavioural patterns. Since the evolutionary interests of men and women often diverge and the two sexes play different roles in social groups (Low 2015), elucidating the possible demographic drivers of sex-specific altruism is key to illuminate human sociality.

Here, we develop a kin-selection model to assess the scope for the demography of warfare – in particular, pre-war dispersal and the movements of individuals between groups resulting from victory or defeat in war – to influence overall levels of sex-specific, within-group altruism. We perform two analyses to generate comparative predictions as to which sex is favoured by natural selection to be more altruistic and which sex is favoured to receive more altruism under different demographic conditions. We adopt a behavioural ecological approach to human behaviour (Nettle *et al.* 2013), focusing on the inclusive fitness effects of acting altruistically and assuming – for the purposes of analysis – that these behaviours are controlled genetically. We define altruism broadly as any behaviour that reduces the competitiveness for reproductive opportunities of the actor, but increases the competitiveness of the recipient (Hamilton 1964; West *et al.* 2007). Such altruism may benefit the whole group – for example, remaining vigilant at night to alert groupmates of predatory threats, contributing to the construction of public goods – or be aimed at one or more individuals – for example, helping with foraging or cultivation work, or sharing fitness-enhancing resources, such as food, tools and shelter. By focusing on overall levels of altruism, our analysis can illuminate the role of sex and demography in generating variation in all of these cases. In order to assess the effects of warfare on social behaviours not directly related to warfare itself, we assume that the altruistic behaviours under consideration do not increase the group's probability of engaging in or winning confrontations with other groups.

## Methods

We employ a two-sex kin-selection model of between-group conflict, adapting and expanding a previously developed framework (Lehmann and Feldman 2008; Micheletti *et al.* 2017, 2018) to consider the evolution of altruism. We perform two analyses. In the first analysis, we explore which sex is favoured to be more altruistic by considering altruism, performed by either men or women, that benefits the group as whole. In the second analysis we investigate which sex altruism should be aimed towards, by considering the evolution of four altruistic behaviours: male-to-male altruism, male-to-female altruism, female-to-male altruism and female-to-female altruism. We determine how selection acts on each of these six altruistic traits by conducting kin-selection analyses (Hamilton 1964; Maynard Smith 1964; Taylor 1996; Taylor and Frank 1996; Frank 1997, 1998; Rousset 2004; Taylor *et al.* 2007) (see Supporting Information for full details).

### Model assumptions

Following Lehmann and Feldman (2008) and Micheletti *et al.* (2017, 2018), we consider an infinitely large population subdivided into an infinite number of groups, each with finite numbers *N*_i_ of adults of sex i ∈ {m,f} where m denotes male and f denotes female, and we consider that all groups are equally distant from each other (assumptions shared with Wright's (1931) infinite island model). These are simplifying assumptions that abstract away from some of the complexity that likely characterised ancestral human populations living a hunter-gather lifestyle in the Pleistocene and many small-scale societies until recent times. For example, distance might have influenced how often groups engaged in war or maintained peaceful relations, and conflicts might have been more frequent or intense between groups from different ethnolinguistic communities (Fry 2013). However, these assumptions afford mathematical tractability (Servedio *et al.* 2014) and allow the derivation of analytical results regarding the selective pressures surrounding intergroup warfare, as demonstrated by previous work adopting them (Lehmann and Feldman 2008; Micheletti *et al.* 2017, 2018).

### Life cycle

At the beginning of the life cycle, the *N*_f_ women and *N*_m_ men in each group mate in a regime of absolute promiscuity and each woman produces a large number *K*_f_ of daughters and a large number *K*_m_ of sons who mature to become young adults (for simplicity and following Lehmann and Feldman (2008) and Micheletti *et al.* (2017, 2018) we assume non-overlapping generations, so that only young adults, hereafter ‘individuals’, can disperse, be altruistic and then reproduce). Each sex-i individual may then disperse to a randomly chosen group with sex-specific probability *m*_i_. After the dispersal phase, individuals have the opportunity to be altruistic towards their groupmates. In the first analysis, we consider altruism *x*_i_ that is performed by sex i and that benefits both male and female groupmates indiscriminately. In the second analysis, we consider altruism *x*_ij_ that is performed by sex i and that is aimed exclusively towards groupmates of sex j – where i, j ∈ {m,f}. The war phase follows, with each post-dispersal group having the chance to attack another randomly chosen group with probability *a* and be attacked by a third group – again randomly selected – with the same probability *a*, and with attackers winning the war with probability *ω*. This is followed by density-dependent regulation, consisting of scramble competition among same-sex competitors for the *N*_i_ sex-i reproductive spots in each group, with different individuals having potentially different competitiveness weightings, but all successful breeders having the same fertility (i.e. no reproductive skew among breeders). Notice that results for reproductive skew could be recovered by substituting *N*_i_ with the appropriate number of ‘effective’ equal-fertility breeders, and our results hold in this case (see Supporting Information).

In groups that are not attacked or that are attacked and successfully defend themselves, sex-i individuals compete only with sex-i groupmates and have competitiveness *t*_i_, which is termed the individual's intrinsic competitiveness. In groups that are attacked and are defeated by their attackers, sex-i individuals instead compete for reproductive opportunities in their group with both sex-i groupmates and with their sex-i attackers, with the conquered group's sex-i individuals having competitiveness for reproductive opportunities (1 – *σ*_i_) *t*_i_ and the conquering group's sex-i individuals having competitiveness *σ*_i_ *t*_i_. The competitiveness modifier *σ*_i_ is equal to the fraction of sex-i reproductive opportunities in a conquered group seized by conquering sex-i individuals and thus modulates the additional reproductive opportunities afforded by warfare to individuals from victorious groups. Notice that, while the model allows for defeated individuals to be forcefully relocated to the victorious group by their conquerors, it does not allow them to have reproductive success in these groups.

### Altruism by men or women towards all groupmates indiscriminately

In the first analysis, the intrinsic competitiveness *t*_i_ of a sex-i individual is modulated by the altruism *x*_i_ performed by that sex-i individual towards male and female groupmates indiscriminately, by the altruism *x*_i_′ the sex-i individual receives from other sex-i individuals, and by the altruism *x*_j_′ the sex-i individual receives from sex-j individuals in their group, such that 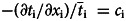 is the competitive cost of being altruistic towards groupmates incurred by the sex-i individual, and 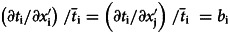 is the marginal increase in competitiveness enjoyed by the sex-i individual as a result of altruism received from groupmates of either sex i or sex j.

### Altruism by men or women towards male or female groupmates exclusively

In the second analysis, the intrinsic competitiveness *t*_i_ of a sex-i individual is modulated by the altruism *x*_ii_ performed by that sex-i individual towards other sex-i individuals, by the altruism *x*_ij_ performed by that sex-i individual towards sex-j individuals, by the altruism *x*_ii_′ the sex-i individual receives from other sex-i individuals, and by the altruism *x*_ji_′ the sex-i individual receives from sex-j individuals in their group, such that 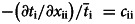 is the competitive cost of being altruistic towards sex-i groupmates incurred by the sex-i individual, 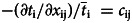 is the competitive cost of being altruistic towards sex-j groupmates incurred by the sex-i individual, 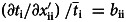 is the marginal increase in competitiveness enjoyed by the sex-i individual as a result of altruism received from sex-i groupmates, and 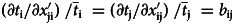 is the marginal increase in competitiveness enjoyed by the sex-i individual as a result of altruism received from sex-j groupmates.

## Results

We first investigate which sex is favoured to perform more altruism, focusing on altruistic behaviours that are uniformly beneficial for the group, that is they are not targeted towards a specific sex of groupmates. Examples of such behaviours include staying on guard to alert groupmates about predators or sharing resources equally among all individuals in the group. Analysing the model, we find that natural selection – including both direct and indirect (i.e. kin selection) effects (Hamilton 1964; Maynard Smith 1964) – favours an individual of sex i ∈ {m,f} to increase altruism that benefits male and female groupmates indiscriminately when:1
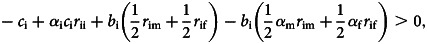
where *c*_i_ is the marginal cost incurred by the sex-i individual on account of their altruism towards their groupmates; *b*_i_ is the marginal benefit enjoyed by the recipient of the altruistic act performed by the sex-i individual; *r*_ij_ = (1 – *m*_i_)(1 – *m*_j_)*r*_x_ is the genetic relatedness between groupmates of sex i and of sex j (with i, j ∈ {m,f} and *r*_x_ being relatedness between individuals born in the same group; see Supporting Information); and *α*_i_ = 1 – 2*aωM*_i_ is the extent to which individuals of sex i compete for reproductive opportunities with same-sex groupmates – i.e. ‘locally’ – as opposed to same-sex individuals in other groups – i.e. ‘globally’ (such that *α*_i_ = 1 is fully local competition and *α*_i_ = 0 is fully global competition).

The scale of competition coefficient *α*_i_ is modulated by a quantity *M*_i_ = (1 – *σ*_i_)*σ*_i_, which describes the extent to which reproductive opportunities in a conquered group are obtained by a mixture of individuals from the winning and the defeated groups (‘admixture’, Micheletti *et al.* 2017; see also Methods). For example, consider a case in which warfare is a purely male domain and thus leads to men from victorious groups reproducing in defeated groups, while women only compete in their home group (*σ*_m_ > *σ*_f_ = 0). This will result in higher male than female admixture (i.e. male-biased admixture, *M*_m_ > *M*_f_ = 0), and thus men will be the more globally competing sex and women the more locally competing sex (*α*_m_ < *α*_f_ = 1). If instead women compete and reproduce in defeated groups to some extent, it can be possible for admixture to be female-biased and, consequently, for women to be competing more globally then men.

The inclusive fitness interpretation of condition (1) is that, by increasing their level of altruism towards groupmates, an individual of sex i incurs: a direct-fitness cost (first term) owing to a –*c*_i_ loss of competitiveness for reproductive opportunities; an indirect-fitness benefit (second term) owing to a corresponding relaxation of kin competition in their own sex, as *c*_i_ reproductive opportunities become available to sex-i individuals who are competing kin with probability *α*_i_ and who are related to the focal individual by *r*_ii_; an indirect-fitness benefit (third term) owing to a *b*_i_ increase in competitiveness for reproductive opportunities for groupmates who, with probability ½, are men related to the focal sex-i individual by *r*_im_ or, with the same probability ½, are women related to the focal sex-i individual by *r*_if_; an indirect-fitness cost (fourth term) owing to a corresponding increase in kin competition, as fewer reproductive opportunities (–*b*_i_) are available for male groupmates (or female groupmates) who are derived from the same group – and are thus competing kin – with probabilities *α*_m_ (or *α*_f_) and who are related to the focal individual by *r*_im_ (or *r*_if_). Note that condition (1) holds even when individuals gain a direct benefit from performing the behaviour (*c*_i_ < 0), in which case this constitutes mutual benefit rather than altruism (West *et al.* 2007; the results of the present analysis thus hold for cooperative behaviours in general, i.e. including both altruism and mutually beneficial cooperation).

Consideration of condition (1) reveals that the demography of warfare can generate incentives for within-group altruism under broad conditions. Specifically, altruism can be favoured whenever warfare exports at least a fraction of male or female competition for reproductive opportunities from winning groups to defeated groups (*α*_m_ ≠ 1 and/or *α*_f_ ≠ 1). This ensures that the inclusive fitness benefit obtained by acting altruistically towards groupmates (third term in condition (1)) is not exactly cancelled by the associated inclusive fitness cost owing to an increase in kin competition (fourth term), as would happen in populations with limited dispersal in the absence of a mechanism decoupling these two effects (Taylor 1992; reviewed in Lehmann and Rousset 2010).

Furthermore, condition (1) can be rearranged into the form *c*_i_/*b*_i_ < *A*_i_, where2
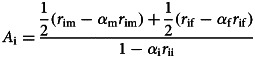
is the ‘potential for altruism’ by members of sex i towards their groupmates (cf. Gardner 2010). This approach separates the cost and benefit functions (*c*_i_/*b*_i_; left-hand side of the rearranged condition) from demographic effects on altruism (the ‘potential’ *A*_i_; right-hand side). By analysing potentials for altruism, we can focus on assessing the effects of sex-specific demography on altruism – the aim of the present study – independently of the details of the cost and benefits functions.

**Figure 1.**
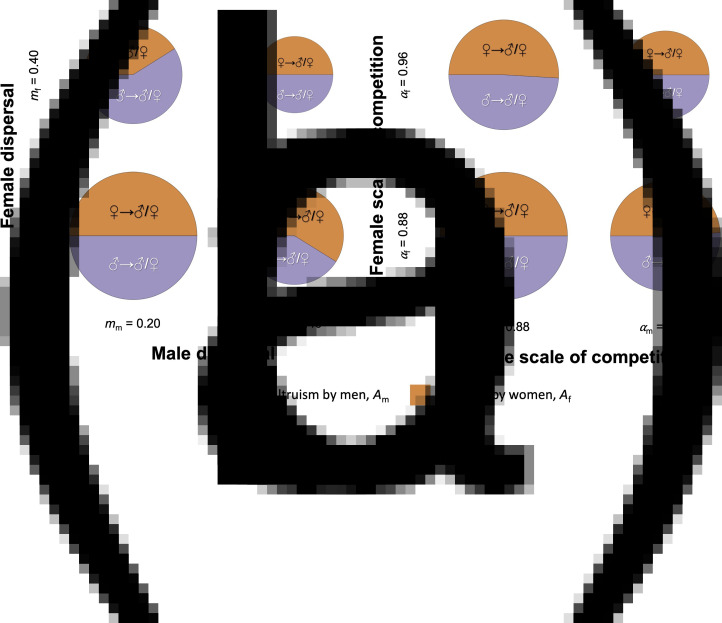
Male and female altruism towards groupmates of either sex. Each pie chart shows potential for altruism by men towards groupmates (purple) vs potential for altruism by women towards groupmates (brown) and the sum of the two potentials (diameter of pie chart) – for low and high values of male and female dispersal rates (a), or for low and high values of male and female scale of competition (b). Other parameter values: (a and b) *N*_m_ = *N*_f_ = 3, *a* = 0.5, *ω* = 0.5; (a) *σ*_m_ = *σ*_f_ = 0.4 leading to *M*_m_ = *M*_f_ = 0.24 and *α*_m_ = *α*_f_ = 0.88; (b) *m*_m_ = *m*_f_ = 0.1, *σ*_i_ = 0.4 leading *M*_i_ = 0.24 (for *α*_i_ = 0.88) and *σ*_i_ = 0.2 leading *M*_i_ = 0.16 (for *α*_i_ = 0.96).

Consideration of the potential for altruism above (equation 2) reveals two key results regarding which sex is favoured to be more altruistic. First, all else being equal, the sex that disperses at a lower rate has a greater potential for altruism than the more dispersing sex (see Figure 1a). For example, if dispersal is female-biased (*m*_m_ < *m*_f_), we predict that men will be more altruistic than women (*A*_m_ > *A*_f_), all else being equal. This prediction arises because, since they disperse less, men are more related to groupmates of either sex than are women (*r*_mm_ > *r*_mf_ > *r*_ff_) and thus reap greater benefits from being altruistic. Second, all else being equal, the sex that – as a result of warfare – competes more locally for reproductive opportunities has a higher potential for altruism towards their groupmates than the more globally competing sex (see Figure 1b). For example, if men from winning groups compete in defeated groups more than women – that is, women compete more locally (*α*_m_ < *α*_f_) – then we predict that women will be more altruistic than men (*A*_m_ < *A*_f_), all else being equal. This prediction arises because, as women compete with other related women more often than men, they reap greater benefits by reducing kin competition through altruistic behaviours than men do (second term in condition 1). In addition, we find that, with increasing values of frequency of war (*a*), levels of potentials for altruism either increase or do not change, but they never decrease (that is, d*A*_i_/d*a* ≥ 0).

We have delineated the conditions under which men and women can be favoured to act altruistically to the benefit of all individuals in their group. We now consider cases in which altruistic behaviours can be targeted at individuals of exclusively one sex – and ask whether such altruism should be directed towards male or female groupmates. Analysing the model, we find that natural selection – including both direct and indirect (i.e. kin selection) effects (Hamilton 1964; Maynard Smith 1964) – favours an individual of sex i to increase their altruism towards groupmates of sex j (where i, j ∈ {m,f}) when:3
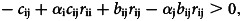
where *c*_ij_ is the marginal cost incurred by the sex-i individual on account of their altruism towards groupmates of sex j and *b*_ij_ is the marginal benefit enjoyed by the sex-j recipient of the altruistic act performed by the sex-i individual.

Condition (3) can be rearranged into the form *c*_ij_/*b*_ij_ < *A*_ij_, where4
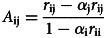
is the potential for altruism by members of sex i towards groupmates of sex j. Notice that, if i = j, *A*_ij_ is the potential for altruism towards the same sex whereas, if i ≠ j, *A*_ij_ is the potential for altruism towards the opposite sex.

**Figure 2.**
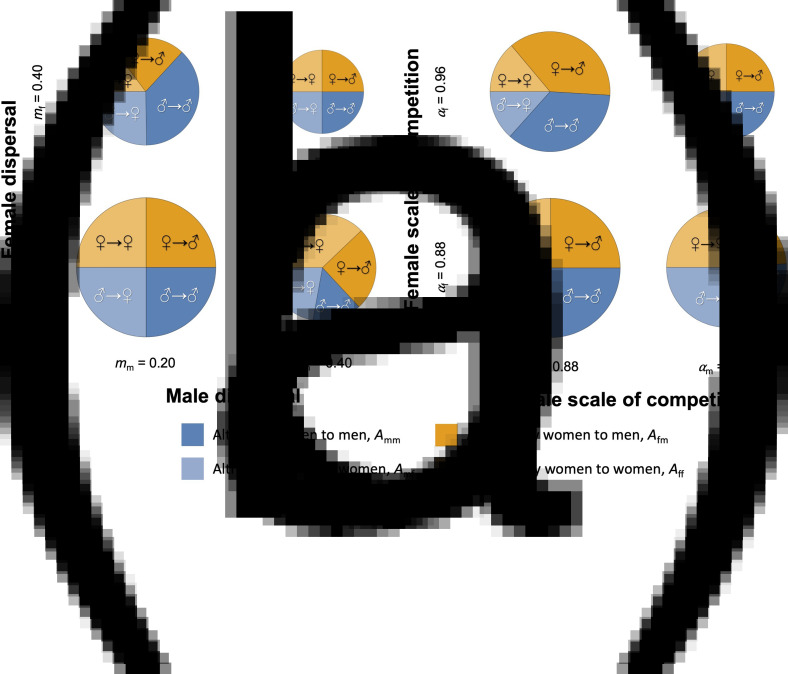
Male-to-male, male-to-female, female-to-male and female-to-female altruism. Each pie chart shows potential for altruism by men towards male groupmates (dark blue) vs potential for altruism by men towards female groupmates (dark orange) vs potential for altruism by women towards male groupmates (light blue) vs potential for altruism by women towards female groupmates (light orange) and the sum of the four potentials (diameter of pie chart) – for low and high values of male and female dispersal rates (a), or for low and high values of male and female scale of competition (b). Other parameter values: (a, b) *N*_m_ = *N*_f_ = 3, *a* = 0.5, *ω* = 0.5; (a) *σ*_m_ = *σ*_f_ = 0.4 leading to *M*_m_ = *M*_f_ = 0.24 and *α*_m_ = *α*_f_ = 0.88; (b) *m*_m_ = *m*_f_ = 0.1, *σ*_i_ = 0.4 leading to *M*_i_ = 0.24 (for *α*_i_ = 0.88) and *σ*_i_ = 0.2 leading to *M*_i_ = 0.16 (for *α*_i_ = 0.96).

Consideration of the potential for altruism immediately above (equation 4) reveals two key results. First, all else being equal, both sexes have a greater potential for altruism towards the sex that disperses less (see Figure 2a). For example, in the case of female-biased dispersal (*m*_m_ < *m*_f_), we predict that men receive more altruism (*A*_mm_ < *A*_mf_ and *A*_fm_ < *A*_ff_), all else being equal. This is because, in this situation, men are more related to groupmates of both sexes than are women (*r*_mm_ > *r*_mf_ > *r*_ff_, as a result of the bias in dispersal) and thus the benefits reaped by actors of both sexes from being altruistic towards men are higher. Second, both sexes have a higher potential for altruism towards members of the sex that competes more globally, all else being equal (see Figure 2b). Considering again an example in which men compete relatively more globally and women relatively more locally (*α*_f_ > *α*_m_), we predict that men will receive more altruism from groupmates of both sexes (*A*_mf_ < *A*_mm_ and *A*_ff_ < *A*_fm_). This is because, as men compete more often with non-relatives, the indirect cost of increased kin competition is lower when helping them than when helping women, for both sexes (fourth term in condition 3). Notice how, in this example, men competing more globally and women more locally leads to women helping groupmates more than men, but both sexes biasing their help more towards men than women in their group (Figures 1b and 2b). In addition, we find that – while higher numbers of reproductive women and men in a group depress relatedness and result in lower potentials for altruism – differences in the numbers of female and male breeders (*N*_m_ ≠ *N*_f_) do not result in sex differences in potential for altruism (*A*_mf_/*A*_mm_ and *A*_ff_/*A*_fm_ are constants with respect to *N*_m_ and *N*_f_; see Supporting Information).

**Figure 3.**
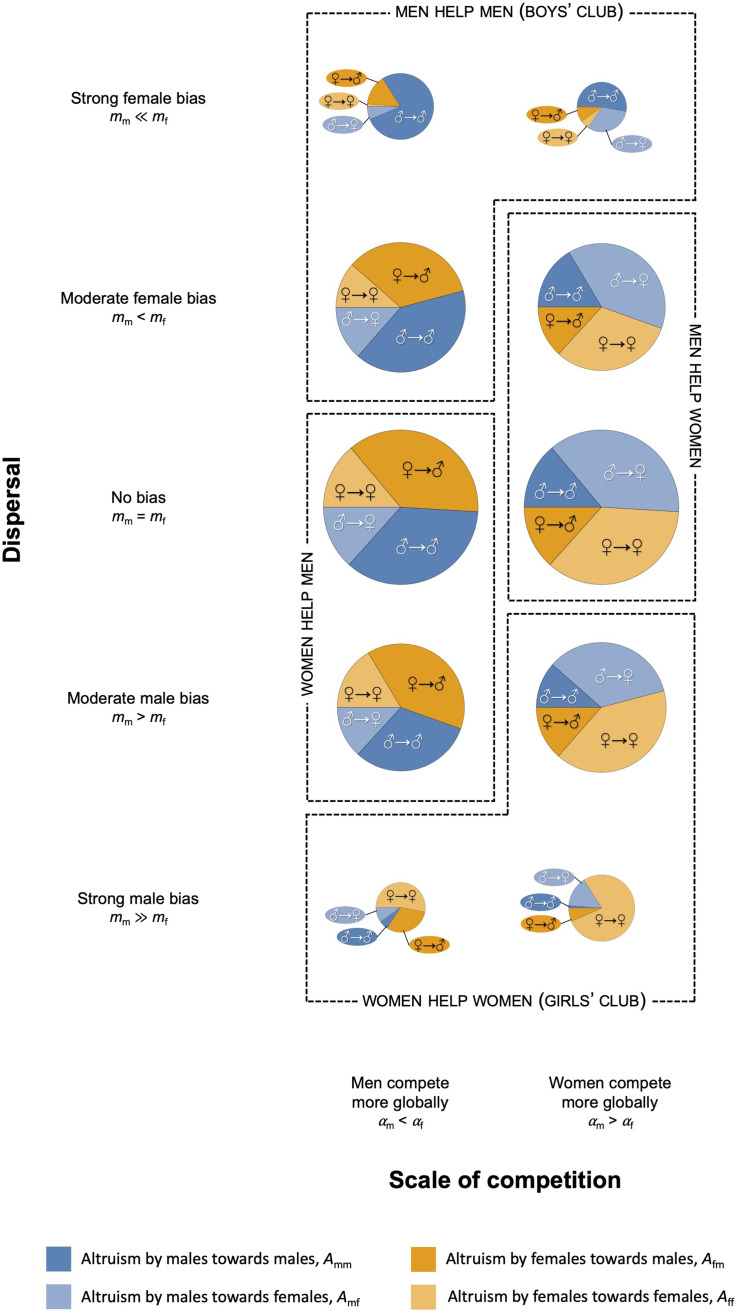
Patterns of altruism as a function of dispersal and the scale of competition. Each pie chart shows potential for altruism by men towards male groupmates (dark blue) vs potential for altruism by men towards female groupmates (dark orange) vs potential for altruism by women towards male groupmates (light blue) vs potential for altruism by women towards female groupmates (light orange) and the sum of the four potentials (diameter of pie chart) – for different combinations of male and female dispersal rates and male and female scale of competition coefficients. Other parameter values: *N*_m_ = *N*_f_ = 3, *a* = 0.5, *ω* = 0.5, *σ*_m_ = 0.4 and *σ*_f_ = 0.1 (‘men compete more globally’, *M*_m_ = 0.24, *M*_f_ = 0.09, *α*_m_ = 0.94 and *α*_f_ = 0.9775), *σ*_m_ = 0.1 and *σ*_f_ = 0.4 (‘men compete more globally’, *M*_m_ = 0.09, *M*_f_ = 0.24, *α*_m_ = 0.9775 and *α*_f_ = 0.94), *m*_m_ = 0.1 and *m*_f_ = 0.8 (‘strong female bias’), *m*_m_ = 0.1 and *m*_f_ = 0.2 (‘moderate female bias’), *m*_m_ = 0.1 and *m*_f_ = 0.1 (‘no bias’), *m*_m_ = 0.2 and *m*_f_ = 0.1 (‘moderate male bias’), *m*_m_ = 0.8 and *m*_f_ = 0.1 (‘strong male bias’).

Moreover, we find that the effects of sex biases in dispersal and the scale of competition – the two key modulators of altruism identified above – may reinforce each other or act in different directions, influencing both who helps and who receives help. Where these two forces act in different directions, who helps more and who receives more help may depend on the exact combination of demographic parameter values (see Supporting Information). It is nonetheless possible to identify four broad patterns of sex-specific altruism. If dispersal is strongly female-biased, men are more altruistic and also receive more help than women, independently of sex differences in the scale of competition. The same configuration is obtained with a moderate male bias in dispersal and men competing more globally. Societies with these demographies follow a ‘boys’ club’ or ‘men help men’ system, where altruism is mostly by and for men. Conversely, if dispersal is strongly male-biased, women are more altruistic and also receive more help than men, again independently of sex differences in the scale of competition. The same pattern is obtained with a moderate male bias in dispersal and women being the more globally competing sex. Societies with these demographies follow a ‘girls’ club’ or ‘women help women’ system, where altruism is mainly a female affair. In the other two configurations the sexes of the predominant helper and helped differ. Women are more altruistic than men, but men receive more altruism when dispersal is moderately male-biased or unbiased, and when men compete more globally (‘women help men’ societies). Instead, men are more altruistic than women, but women receive more altruism when dispersal is unbiased or moderately female-biased, and when women compete more globally (‘men help women’ societies; see Figure 3).

## Discussion

Recent years have seen great interest in the possible coevolution of between-group violence and within-group altruism, but the potential for warfare – via its impact on demography – to influence human social behaviours more widely and in sex-specific ways has been neglected. Here we performed an inclusive fitness analysis to determine how selection acts on altruistic behaviours performed by men and women – either for the benefit of all groupmates or targeted at males or females specifically – in populations experiencing intergroup conflict. We found that warfare can promote within-group altruism, even if this altruism does not improve the group's performance in war. Furthermore, we found that the degree of altruism may crucially depend upon the sex of the altruist and the recipient, and upon two sex-specific demographic parameters: dispersal and the scale of competition.

Our analysis has revealed that warfare – through its effect on demography – can promote altruism, broadly defined (Hamilton 1964; West *et al.* 2007). This is because, as a result of intergroup conflict, individuals from victorious groups compete for reproductive opportunities with non-relatives in defeated groups to some extent and, in this way, decrease harmful competition between kin that would have otherwise inhibited the evolution of altruism. This result contributes to clarifying the role of demography in promoting cooperation and altruism. In a much-celebrated paper, Taylor (1992) showed that, in a population subdivided into groups with limited dispersal, altruism could not spread, because the benefit obtained by helping groupmates was exactly cancelled by the kin competition cost generated by these behaviours (see also Frank 1985, 1986a, b; Bulmer 1986 for an earlier, analogous result for sex allocation). Subsequent work showed that a number of mechanisms could lead to the decoupling of the effects of dispersal on relatedness and kin competition, allowing for the evolution of altruism (reviewed in Lehmann and Rousset 2010). One such avenue involves individuals dispersing together with other groupmates, a mode of migration first discussed by Haldane (1932) in his classic ‘tribe splitting’ model of altruism and now termed ‘budding dispersal’ (Gardner and West 2006; see also Goodnight 1992). In this way, members of a group can be competing for resources with non-relatives to some extent – reducing kin competition – while staying in close proximity to their kin – maintaining high relatedness (Gardner and West 2006; Lehmann *et al.* 2006).

In Lehmann and Feldman's (2008) model of warfare, intergroup conflict allows for the decoupling of relatedness and competition: when a group is defeated in war, winning males compete for reproductive opportunities with losers, thus maintaining relatedness at a high level while reducing kin competition. This mechanism is distinct from budding dispersal as previously described – because competition involves a mix of individuals from two different groups rather than occurring between buds – but presents a strong analogy with it – as movement to other groups occurs in association with groupmates rather than individually. Owing to this demographic effect of warfare, natural selection can favour men to express costly altruistic belligerence and bravery behaviours that increase the group's probability of engaging in and winning a war (Lehmann and Feldman 2008). Here, we showed that this insight holds for all altruistic behaviours expressed by either men or women – not only those that directly impact on a group's success in warfare and are expressed exclusively by men. In this respect, our result also illuminates Haldane's (1932) insight that within-group altruism could only have evolved genetically in early human societies if groups were small and composed of related individuals – a combination that, he argued, could have only been achieved by human groups splitting periodically. Our analysis suggests that warfare, with the movement of individuals to defeated groups after victory, offers a mechanism for this splitting to occur. In this way, related individuals, who are more likely to carry genes encoding a given altruistic behaviour, continue to be in close proximity to each other generation after generation – and altruism has the possibility to spread.

Moreover, we showed that sex biases in two factors in the demography of warfare – dispersal and the scale of competition – may drive sex-specific patterns of altruism. Firstly, we found that the sex that disperses less is favoured both to be more altruistic and to receive more altruism. It is important to underline that, following Lehmann and Feldman (2008) and Micheletti *et al.* (2017, 2018), we have assumed that altruistic behaviours are performed after dispersal (the same assumption is made in other studies investigating the dispersal–altruism interplay outside the context of warfare; e.g. Johnstone and Cant 2008). This means that members of the more philopatric sex are more related to their groupmates, but do not experience higher local competition. If, instead, altruistic behaviours were performed before dispersal, then members of the less-dispersing sex would experience relatively higher local competition – which could possibly outweigh the benefits of being altruistic towards highly related groupmates. Our major qualitative results about sex-biases in altruism would still hold in this case, although overall the benefits of altruism would need to be higher for the behaviour to be selected.

Secondly, we revealed that sex differences in patterns of altruism can be driven by biases in the scale of competition, that is the extent to which men and women from victorious groups compete with non-relatives in defeated groups. Specifically, the sex that competes more locally is favoured to be more altruistic, whereas the sex that competes more globally is favoured to receive more altruism, from members of both sexes. For example, consider a scenario in which most men in a defeated group are excluded from competing for reproduction by winning men, while both winning and defeated women compete for marriage and resources in that group. In this case, warfare results in competition being relatively more global for women than for men. Under these conditions, men are favoured to be more altruistic than women, because they have more to gain by reducing kin competition within their sex through altruism. At the same time, both sexes are favoured to behave more altruistically towards women. This is because, although altruism can improve the competitiveness of both female and male groupmates, the altruist's male groupmates tend to compete for reproductive opportunities against each other (such that one male relative's gain is another male relative's loss). On the other hand, the altruist's female groupmates are more likely to compete for reproductive opportunities against unrelated individuals in other social groups. For this reason, the returns in terms of inclusive fitness benefits are higher when helping the sex that competes more globally.

Our aim has been to capture how demography modulates sex-specific altruism in populations experiencing warfare. To this end, we have focused on the roles of dispersal and the scale of competition, whilst assuming that both sexes are equally capable of helping, that is they experience the same costs and provide the same benefits to their recipients. However, if the sexes did differ in the costs and benefits of altruism, then this – fairly trivially – would be expected to drive sex differences in their altruistic behaviour. For example, if one sex were less effective in helping – i.e. providing a lower benefit – this would lead to a higher cost-to-benefit ratio and thus a lower level of altruism than for the more effective sex. If instead the two sexes were equally effective in helping, but one incurred lower costs associated with it, this would lead to a lower cost-to-benefit ratio and thus a higher level of altruism than for the sex experiencing more substantial costs. In addition, we have not considered altruism that is targeted at specific individuals on the basis of kin recognition, which is likely to be an important modulator of human social behaviour. Incorporating kin recognition need not affect the overall level of altruism enacted and received by each sex (GS Faria and A Gardner, ‘Does kin discrimination promote cooperation?’, under review), but would enable investigation of individual-level variation in addition to the between-sex and between-population variation that we have considered in the present analysis.

Furthermore, we found that the effects of sex biases in dispersal and competition can reinforce each other or act in opposing directions, resulting in four broad patterns of sex-specific altruism which we term ‘boys’ club’, ‘women help men’, ‘men help women’ and ‘girls’ club’ (summarised in Figure 3). However, which of these patterns can we expect to find in current human populations? We suggest that two different timescales can be potentially relevant, and we explore them in turn.

It might be that demographic asymmetries need to persist for very long times – on the order of millions of years – to have an influence on patterns of sex-specific altruism. This would be the case if the traits under consideration were genetically inherited, or if we were considering genetic components contributing to them. In this circumstance, the demographic patterns relevant for our study would be those of ancestral human populations. Sex-biased dispersal in ancestral humans has been greatly debated in recent years (see Box 1). If, as some argue (Ember 1975, 1978; Manson and Wrangham 1991; Seielstad *et al.* 1998; Chapais 2008), female-biased dispersal was dominant early in our evolutionary history and persisted rather uniformly until recently, then we would expect to find the altruism patterns revealed by our model under this dispersal regime. For example, had this migration pattern been accompanied by men competing more globally because of warfare, this would have led to ‘boys’ club’ systems where men both helped more and received more help. If instead, as others suggest (Marlowe 2004; Wilkins and Marlowe 2006; Hill *et al.* 2011; Sugiyama 2017), dispersal was unbiased in ancestral human populations, then – with respect to genetic components – we would expect no sex differences in altruism.

Box 1. Sex-specific dispersal in ancestral humans

Recent years have witnessed a heated debate surrounding patterns of sex biased dispersal in ancestral human populations, living a hunter-gatherer lifestyle in the Pleistocene (Marlowe, 2004; Wilkins and Marlowe, 2006; Sugiyama, 2017). Here, we summarise the current state of the evidence.

Firstly, extant great apes – chimpanzees and bonobos, our closest living relatives, and gorillas – are all characterised by female-biased dispersal (Lawson Handley and Perrin 2007), which has led some to argue that ancestral humans too are likely to have followed this pattern (e.g. Manson and Wrangham 1991; Chapais 2008; but see Sugiyama 2017). Secondly, population genetics studies have examined variation in mitochondrial DNA (maternally inherited) and the non-recombining region of the Y-chromosome (paternally inherited), offering contrasting dispersal estimates. In two global comparisons, Seielstad *et al.* (1998) found evidence of higher female dispersal, but Wilder *et al.* (2004) found no such bias. Wilkins and Marlowe (2006) argue that these results stem from differences in the sampling scheme adopted, with the study finding unbiased dispersal being more reflective of pre-agricultural societies and the study finding a female bias capturing residential patterns that emerged after the spread of agriculture. Thirdly, dispersal in extant hunter-gatherers can be informative as these societies are considered the best available models for pre-agricultural populations (Marlowe, 2005). Hunter-gatherer societies have long been characterised as mostly patrilocal (i.e. women moving to the husband's group; Ember 1975, 1978; Foley 1995). However, recent work has shown that hunter-gatherers move frequently between bands and spouses can move to either the husband's or the wife's band: a system best described as ‘multilocal’ or ‘bilocal’ (Marlowe 2004; Hill *et al.* 2011). However, this does not mean that dispersal is necessarily unbiased. For example, in a study of 32 extant hunter-gather societies, Hill *et al.* (2011) found more co-residing brothers than sisters in 17 societies, the opposite pattern in three societies, and no bias in the remaining 12.

Overall, notwithstanding the great developments in these three research avenues, it is still unclear what pattern of dispersal was followed by our Pleistocene ancestors. It is possible that dispersal was on average female-biased throughout our history, becoming more pronounced with the advent of agriculture. Alternatively, it may have been unbiased during the Pleistocene and became female-biased after the agricultural revolution, in association with patrilocal residence rules.

On the other hand, it might be that more recent changes in patterns of dispersal – on the order of centuries or millennia – can result in differences in sex-specific altruism between populations. This would be the case if the altruistic traits under investigation were culturally inherited, or if we were considering culturally inherited contributors to such behaviours. Notice that, since our model identifies men's and women's inclusive fitness interests, our *qualitative* predictions as to sex differences in altruistic behaviours would still be relevant if the traits under question were cultural variants selected on the basis of their effects on the biological fitness of their carrier (‘type-1 cultural selection’, Birch 2017; see also Mace 2014). Therefore, in such a case, we would expect societies with different post-marital residence rules – cultural norms mediating dispersal in humans that can change relatively rapidly (Jordan *et al.* 2009; Opie *et al.* 2014; Ji *et al*. 2016) – to show the patterns predicted by our model for the corresponding sex biases in dispersal.

In any of these two cases, empirical studies investigating sex differences in cooperation could offer some support for the results of our study. The potential for such differences has been studied extensively employing economic games in the laboratory, with contrasting results: one metanalysis revealed that interactions between men are more cooperative than interactions between women (Balliet *et al.* 2011), whereas another found that women are more cooperative than men (Engel 2011). However, these studies – performed almost exclusively with WEIRD (western, educated, industrialised, rich, and democratic; Henrich *et al.* 2010) participants – do not consider ecological variables and thus cannot illuminate the role of demographic effects on altruism. Instead, our predictions are best tested in the field, specifically in small-scale societies that vary in dispersal patterns and that currently engage in – or have a recent history of – intergroup conflict.

Two studies investigating cooperation in agro-pastoral societies in South-Western China with economic games (Gong *et al*. 2015; Wu *et al.* 2015) might offer some support for the demographic effects identified in our model. These societies – Pumi, Han, Yi, Amdo, Khampa, Mosuo and Zhaba – share similar ecologies while differing substantially in post-marital residence rules, the cultural norms regulating dispersal in human societies (Jordan *et al.* 2009). In addition, blood feuds and cattle raiding are now very much limited by state policing but were common in most of these groups until recently (Cai 2001; Yeh 2003; Pirie 2005; Harrell 2011; Shi 2018) and it is reasonable that they might still influence cultural norms and actual behaviour. Wu *et al.* (2015) showed that men are more cooperative and/or altruistic than women in both duolocal (neither sex migrates, unbiased dispersal) and patrilocal (female-biased dispersal) populations. At the same time, they found lower levels of cooperation when more men than women participate in the games (Wu *et al.* 2015), which might indicate that both sexes are more incentivised to be altruistic towards women than towards men. Our results suggest that this pattern might have been driven by warfare: men being more altruistic and women receiving more altruism is the outcome we obtain under unbiased or moderately female-biased dispersal – as observed in these societies – and with women competing more globally than men as a result of war (‘men help women’, see Figure 3). To our knowledge, patterns of post-war competition in defeated groups are unknown for these societies, but could be possibly inferred from oral reports and historical documents regarding intergroup conflict (an example of how greater female competition might be realised as a result of war is discussed above).

Moreover, Gong *et al.* (2015) found that duolocal Mosuo men give more than women in the Dictator game, and they also give more than patrilocal Yi men. Given that these societies show unbiased or moderately female-biased dispersal, these results might again be in line with our ‘men help women’ configuration (Figure 3), but the study does not test for the effect of receiver's sex. Furthermore, the finding that altruistic giving is lower in patrilocal populations (Gong *et al.* 2015), might result from higher dispersal rates in these groups leading to lower relatedness – as predicted by our model. Interestingly, Wu *et al.* (2015) found the opposite pattern: higher dispersal societies show more altruism. Additional work, both theoretical and empirical, is clearly needed to disentangle the effects of dispersal on relatedness and kin competition and determine how the two might interact to influence social behaviours in human populations. Elucidating demographic factors – and the within-group or between-group processes influencing it, including warfare – promises to be a fruitful avenue.

## Supplementary material

For supplementary material accompanying this paper visit http://dx.doi.org/10.1017/ehs.2020.5. click here to view supplementary material
