# Supplementary material for: The demography of human warfare can drive sex differences in altruism
Source: Evol Hum Sci. 2020 Feb 20;2:e7. doi: 10.1017/ehs.2020.5 (PMC10427324; doi:10.1017/ehs.2020.5)
Supplement: Supplementary file 1 [file S2513843X20000055sup001.pdf]

Supporting Information for

The demography of human warfare can drive sex differences in altruism

Alberto J. C. Micheletti, Graeme D. Ruxton & Andy Gardner

Contents

1 **Preamble** 1

2 **Fitness** 2

3 **Altruism to unspecified sex – marginal fitness** 4

4 **Altruism to same sex – marginal fitness** 7

5 **Altruism to opposite sex – marginal fitness** 9

6 **Consanguinity and relatedness** 12

7 **Demonstrations** 13

8 **Additional references** 16

1 Preamble

Here, we adapt an existing model of warfare (Lehmann and Feldman 2008; Micheletti *et al.* 2017; 2018) to investigate the evolution of sex-specific altruism – i.e. a behaviour that results in a decrease in competitiveness for the altruist and an increase in competitiveness for the recipient (Hamilton 1964; West *et al.* 2007) – in the context of the demography of war (i.e. pre-war dispersal and the movements of individuals between groups resulting from victory or defeat in war). We adopt the life cycle described in Lehmann and Feldman (2008) and Micheletti *et al.* (2017; 2018), but we consider the probability that a group attacks another one and the probability that the attacking group wins the war as non-evolving parameters. We consider six altruistic traits: altruism by males or females towards groupmates of unspecified sex (see **3 Altruism to unspecified sex – marginal fitness**), male-to-female altruism, female-to-male altruism (see **4**

Altruism to opposite sex – marginal fitness), male-to-male altruism, female-to-female altruism (see **5 Altruism to same sex – marginal fitness**).

2 Fitness

We first derive the absolute fitness of a focal young adult individual of sex i $\in$ {m,f}. With probability $1 - m_\mathrm{i}$, the focal young adult of sex-i does not disperse from the focal group. In every generation, each post-dispersal group can attack one randomly-chosen group. With probability 1 – a, the focal group is not attacked by another group. In this case the focal sex-i young adult competes for N_i breeding spots with $N_\mathrm{f}\ K_\mathrm{i}$ sex i young adults, where N_f is the number of adult females in a group, whereas K_i is the number of sex-i offspring produced by each adult female and is assumed to be large. Notice that a fraction $1 - m_\mathrm{i}$ of these sex i young adults originated from the focal group, while the remaining proportion m_i migrated from another group (the dispersal terms cancel out). The focal sex i young adult has intrinsic competitiveness for reproductive spots t_i and his groupmates t_i'. Therefore, the probability that a focal sex i young adult secures a breeding spot is $\frac{N_\mathrm{i} t_\mathrm{i}}{N_\mathrm{f} K_\mathrm{i} t_\mathrm{i}'}$. Alternatively, with probability a, the focal group is attacked and the attacking group loses the war with probability 1 – ω, in which case the probability that a focal sex i young adult secures a breeding spot is again $\frac{N_\mathrm{i} t_\mathrm{i}}{N_\mathrm{f} K_\mathrm{i} t_\mathrm{i}'}$. Otherwise, the attacking group wins the war with probability ω, in which case the focal sex-i young adult competes for $N_\mathrm{i}(1 - \sigma_\mathrm{i})$ breeding spots with $N_\mathrm{f} K_\mathrm{i}(1 - \sigma_\mathrm{i})$ sex-i young adults from their group and $N_\mathrm{f} K_\mathrm{i} \sigma_\mathrm{i}$ sex-i young adults from the attacking group – resulting in a probability $\frac{N_\mathrm{i}(1-\sigma_\mathrm{i}) t_\mathrm{i}}{N_\mathrm{f} K_\mathrm{i}(1-\sigma_\mathrm{i}) t_\mathrm{i}' + N_\mathrm{f} K_\mathrm{i} \sigma_\mathrm{i} \bar{t}_\mathrm{i}}$ that the focal sex i young adult obtains a reproductive spot. In addition, the focal group may attack one other group. With probability 1 – a, this does not happen, and with probability $a(1 - \omega)$, they attack but lose the war: in either case, the focal sex-i young adult does not have access to additional reproductive spots. If instead the focal group attacks another group and wins the war – which occurs with probability $a\omega$, then the focal sex-i young adult has access to an additional $N_\mathrm{i} \sigma_\mathrm{i}$ breeding spots, for which he competes with $N_\mathrm{f} K_\mathrm{i} \sigma_\mathrm{i}$ sex-i young adults from their group and $N_\mathrm{f} K_\mathrm{i}(1 - \sigma_\mathrm{i})$ sex-i young adults from the conquered group – resulting in a probability $\frac{N_\mathrm{i} \sigma_\mathrm{i} t_\mathrm{i}}{N_\mathrm{f} K_\mathrm{i} \sigma_\mathrm{i} t_\mathrm{i}' + N_\mathrm{f} K_\mathrm{i}(1-\sigma_\mathrm{i}) \bar{t}_\mathrm{i}}$ that the focal sex i young adult obtains a breeding spot.

Alternatively, the focal sex-i young adult disperses from the natal group with probability m_{i} to a randomly chosen group. In this case, the probabilities that a focal individual of sex i obtains a reproductive spot are identical to the case described above in which they do not disperse. Notice that the dispersal terms cancel out and thus do not appear in the fitness function (but dispersal has an impact on relatedness, see **6 Consanguinity and relatedness**). Therefore, the fitness of a focal sex-i young adult is given by:

$$w_{\mathrm{i}} = \left((1-a)\frac{t_{\mathrm{i}}}{t_{\mathrm{i}}'} + a\left((1-\omega)\frac{t_{\mathrm{i}}}{t_{\mathrm{i}}'} + \omega \frac{(1-\sigma_{\mathrm{i}})t_{\mathrm{i}}}{(1-\sigma_{\mathrm{i}})t_{\mathrm{i}}' + \sigma_{\mathrm{i}}\bar{t}_{\mathrm{i}}} \right) + a\omega \frac{\sigma_{\mathrm{i}} t_{\mathrm{m}}}{\sigma_{\mathrm{i}} t_{\mathrm{i}}' + (1-\sigma_{\mathrm{i}})\bar{t}_{\mathrm{i}}} \right) \frac{N_{\mathrm{i}}}{N_{\mathrm{f}} K_{\mathrm{i}}} \qquad \text{[A2.1]}$$

The average fitness of a sex-i young adult in the population is obtained by setting $t_{\mathrm{i}} = t_{\mathrm{i}}' = \bar{t}_{\mathrm{i}}$ and is given by $\bar{w}_{\mathrm{i}} = \frac{N_{\mathrm{i}}}{N_{\mathrm{f}} K_{\mathrm{f}}}$. The relative fitness of the focal sex-i young adult is given by $W_{\mathrm{i}} = \frac{w_{\mathrm{i}}}{\bar{w}_{\mathrm{i}}}$. The relative fitness of a young adult of unspecified sex in a focal group in a sex-structured population is a weighted average of the relative fitness of male and female individuals and is given by $W = c_{\mathrm{m}} W_{\mathrm{m}} + c_{\mathrm{f}} W_{\mathrm{f}}$, where c_{m} and c_{f} are the class reproductive values for males and females, respectively (Fisher 1930; Price and Smith 1972; Taylor and Frank 1996; Frank 1997; Taylor *et al.* 2007).

2.1 A note about fertility and reproductive skew

Following Lehmann and Feldman (2008) and Micheletti *et al.* (2017, 2018), we consider that N_{f} adult women and N_{m} adult men mate randomly within each group, with all men having the same fertility (each fathering K_{f} daughters and K_{m} sons) and all women having the same fertility (each bearing K_{f} daughters and K_{m} sons) so that there is no reproductive skew. The parameters N_{f} and N_{m} influence the results of the model by determining the probabilities that two juveniles born in the same group share the same mother ($\nu_{\mathrm{f}} = 1/N_{\mathrm{f}}$) and the same father ($\nu_{\mathrm{m}} = 1/N_{\mathrm{m}}$) (see equation [A6.1] in section **6 Consanguinity and relatedness**). Thus, results for reproductive skew could be recovered by substituting in the appropriate 'effective' $N_{\mathrm{f}} = 1/\nu_{\mathrm{f}}$ and $N_{\mathrm{m}} = 1/\nu_{\mathrm{m}}$ values. For example, in the case where there are two adult women breeding per patch, one producing 10% of the total number of juveniles in the group and the other producing 90%, the probability that two juveniles share the same mother would be given by $0.1^2 + 0.9^2 = 0.82$,

and hence the effective number of female breeders would be $N_{\mathrm{f}} = 1/0.82 = 1.22$. Notice that, while our model assumes no reproductive skew, all our qualitative results regarding the effects of biases in dispersal and the scale of competition on patterns of sex-specific altruism hold if reproductive skew is allowed (see points 7-10 in section **7 Demonstrations**).

3 Altruism to unspecified sex – marginal fitness

Consider a locus $\mathcal{G}$, which controls altruism performed by individuals of sex i $\in$\{m,f\} towards groupmates of unspecified sex, and denoted by x_{i}. This trait is expressed only by young adults of sex i, but impacts the competitiveness for breeding spots of individuals both sex i and sex j in their group (where j $\in$\{m,f\}). Let g be the genic value of the focal young adult for this gene, G the breeding value of the focal young adult, G' the breeding value of a randomly-chosen groupmate of the focal young adult, and $\bar{G}$ the average of the population.

Assuming vanishing genetic variation – all breeding values clustered around the mean (Taylor and Frank 1996; Frank 1997) – the direction of natural selection is given by:

$$\frac{\mathrm{d}W}{\mathrm{d}g} = \mathbb{C}_{\mathrm{i}}\frac{\mathrm{d}W_{\mathrm{i}}}{\mathrm{d}g_{\mathrm{i}}} + \mathbb{C}_{\mathrm{j}}\frac{\mathrm{d}W_{\mathrm{j}}}{\mathrm{d}g_{\mathrm{j}}}, \qquad \text{[A3.1]}$$

where $W = \mathbb{C}_{\mathrm{i}}W_{\mathrm{i}} + \mathbb{C}_{\mathrm{j}}W_{\mathrm{j}}$ is the average of relative fitness taken over sex i and sex j classes and $\mathbb{C}_{\mathrm{i}}$ and $\mathbb{C}_{\mathrm{j}}$ are the the class reproductive values for the two sexes (Fisher 1930; Taylor 1996); and with all derivatives evaluated at $g = g_{\mathrm{i}} = g_{\mathrm{j}} = \bar{G}$.

The first derivative in equation [A.3.1] describes the impact of the genic value of a gene drawn from a sex-i young adult on their relative fitness. This can be expanded, employing the chain rule, obtaining:

$$\frac{\mathrm{d}W_{\mathrm{i}}}{\mathrm{d}g_{\mathrm{i}}} = \frac{\partial W_{\mathrm{i}}}{\partial x_{\mathrm{i}}}\frac{\mathrm{d}x_{\mathrm{i}}}{\mathrm{d}G}\frac{\mathrm{d}G}{\mathrm{d}g_{\mathrm{i}}} + \frac{\partial W_{\mathrm{i}}}{\partial x_{\mathrm{i}}'}\frac{\mathrm{d}x_{\mathrm{i}}'}{\mathrm{d}G'}\frac{\mathrm{d}G'}{\mathrm{d}g_{\mathrm{i}}} = \left(\frac{\partial W_{\mathrm{i}}}{\partial x_{\mathrm{i}}}p_{\mathrm{self}} + \frac{\partial W_{\mathrm{i}}}{\partial x_{\mathrm{i}}'}p_{\mathrm{ii}}\right)\gamma\,, \qquad \text{[A3.2]}$$

where $\frac{\partial W_i}{\partial x_i}$ is the impact of the altruistic behaviour performed by the focal sex-i young adult on their fitness, $\frac{\partial W_i}{\partial x_i'}$ is the impact of the altruistic behaviour performed by a random sex-i young adult in the focal group on the fitness of the focal sex-i young adult, $p_{\text{self}} = \frac{\text{d}G}{\text{d}g_i}$ is the consanguinity of the focal sex-i young adult to themselves, $p_{ii} = \frac{\text{d}G'}{\text{d}g_i}$ is the consanguinity between the focal sex-i young adult and a random sex-i young adult in their group, and $\gamma = \frac{\text{d}x_i}{\text{d}G} = \frac{\text{d}x_i'}{\text{d}G'}$ is the correlation between a young adult's phenotype and their breeding value.

The second derivative in equation [A3.2] describes the impact of the genic value of a gene drawn from a young adult of the other sex (sex j) on their relative fitness. Expanding it, again employing the chain rule, reveals an indirect fitness component (there is no direct fitness component as sex-j young adults do not perform the behaviour). In mathematical form:

$$\frac{\text{d}W_j}{\text{d}g_j} = \frac{\partial W_j}{\partial x_i'}\frac{\text{d}x_i'}{\text{d}G'}\frac{\text{d}G'}{\text{d}g_j} = \frac{\partial W_j}{\partial x_i{}'}\, p_{ij}\, \gamma\,, \qquad \text{[A3.3]}$$

where $\frac{\partial W_j}{\partial x_i'}$ is the impact of the altruistic behaviour performed by a random sex-i young adult in the focal group on the fitness of the focal sex-j young adult, $p_{ij} = \frac{\text{d}G'}{\text{d}g_j}$ is the consanguinity between the focal sex-j young adult and a random sex-i young adult in their group, and $\gamma = \frac{\text{d}x_i'}{\text{d}G'}$ is again the correlation between a young adult's phenotype and their breeding value.

Altruism performed by sex i towards individuals of unspecified sex has an impact on both sex-i competitiveness for breeding positions t_i and sex-j competitiveness t_j (see Methods). Therefore: $\frac{\partial W_i}{\partial x_i} = \frac{\partial W_i}{\partial t_i}\frac{\partial t_i}{\partial x_i}$, $\frac{\partial W_i}{\partial x_i'} = \frac{\partial W_i}{\partial t_i}\frac{\partial t_i}{\partial x_i'} + \frac{\partial W_i}{\partial t_i'}\frac{\partial t_i'}{\partial x_i'}$, $\frac{\partial W_j}{\partial x_i'} = \frac{\partial W_j}{\partial t_j}\frac{\partial t_j}{\partial x_i'} + \frac{\partial W_j}{\partial t_j'}\frac{\partial t_j'}{\partial x_i'}$, with $t_i = T_i(y, z)$ where $y = x_i$ and $z = x_i'$, with $t_i' = T_i(y, z)$ where $y = x_i'$ and $z = x_i'$, and with $t_j = t_j' = T_j(x_i')$. We define: $\frac{\partial t_i}{\partial y} = \frac{\partial t_i'}{\partial y} = -\bar{t}_i\, c_i$ and $\frac{\partial t_i}{\partial z} = \frac{\partial t_i'}{\partial z} = \frac{\partial t_j}{\partial x_i'} = \frac{\partial t_j'}{\partial x_i'} = \bar{t}_i\, b_i$. Recipients of altruism are sex i individuals or sex j individuals with the same probability $\frac{1}{2}$. Therefore we obtain: $\frac{\partial W_i}{\partial x_i} = \frac{\partial W_i}{\partial t_i}(-\bar{t}_i\, c_i)$, $\frac{\partial W_i}{\partial x_i'} = \frac{1}{2}\frac{\partial W_i}{\partial t_i'}(-\bar{t}_i\, c_i) + \frac{1}{2}(\frac{\partial W_i}{\partial t_i}\bar{t}_i\, b_i + \frac{\partial W_i}{\partial t_i'}(-\bar{t}_i\, c_i + \bar{t}_i\, b_i))$, $\frac{\partial W_j}{\partial x_i'} =$

$\frac{1}{2}(\frac{\partial Wj}{\partial tj}\bar{t}_j\, b_i + \frac{\partial W_j}{\partial t_j'}\bar{t}_j\, b_i) + \frac{1}{2}\times 0$. Substituting these expressions and equations [A3.2] and [A3.3] into equation [A3.1] we obtain:

$$\frac{\mathrm{d}W}{\mathrm{d}g} = \mathbb{C}_i\left(\frac{\partial W_i}{\partial t_i}(-c_i\bar{t}_i)p_{self} + \left(\frac{1}{2}\frac{\partial W_i}{\partial t_i'}(-c_i\bar{t}_i) + \frac{1}{2}\left(\frac{\partial W_i}{\partial t_i}(b_i\bar{t}_i) + \frac{\partial W_i}{\partial t_i'}(-c_i\bar{t}_i + b_i\bar{t}_i)\right)\right)p_{ii}\right)\gamma + \mathbb{C}_j\left(\frac{\partial W_j}{\partial t_j}b_i\bar{t}_j + \frac{\partial W_j}{\partial t_j'}b_i\bar{t}_j\right)p_{ij}\,\gamma \qquad [A3.4]$$

Population average altruism by sex i towards sex j increases when $\frac{\mathrm{d}W}{\mathrm{d}g} > 0$. Substituting the appropriate derivatives in equation [A3.4], this condition is given by

$$\mathbb{C}_i\left(-c_ip_{self} + \left(\frac{1}{2}\alpha_ic_i + \frac{1}{2}\left(b_i - \alpha_i(-c_i + b_i)\right)\right)p_{ii}\right)\gamma + \mathbb{C}_j\frac{1}{2}\left(b_i - \alpha_jb_i\right)p_{ij}\,\gamma > 0 \qquad [A3.5]$$

If $\mathbb{C}_i = \mathbb{C}_j = \frac{1}{2}$ (as is the case under diploid inheritance, i.e. the case considered here; Fisher 1930; Taylor 1996), this simplifies to:

$$-c_ip_{self} + \alpha_ic_ip_{ii} + b_i\left(\frac{1}{2}p_{ij} + \frac{1}{2}p_{ii}\right) - b_i\left(\frac{1}{2}\alpha_jp_{ij} + \frac{1}{2}\alpha_ip_{ii}\right) > 0 \qquad [A3.6]$$

Dividing by p_{self} to obtain relatedness coefficients ($r_{ii} = \frac{p_{ii}}{p_{self}}$ and $r_{ij} = \frac{p_{ij}}{p_{self}}$; see **6 Consanguinity and relatedness**) and rearranging terms yields:

$$-c_i + \alpha_ic_ir_{ii} + b_i\left(\frac{1}{2}r_{ij} + \frac{1}{2}r_{ii}\right) - b_i\left(\frac{1}{2}\alpha_jr_{ij} + \frac{1}{2}\alpha_ir_{ii}\right) > 0 \qquad [A3.7]$$

Considered that both males and females receive altruism, this can be rewritten more clearly as:

$$-c_i + \alpha_ic_ir_{ii} + b_i\left(\frac{1}{2}r_{im} + \frac{1}{2}r_{if}\right) - b_i\left(\frac{1}{2}\alpha_mr_{im} + \frac{1}{2}\alpha_fr_{if}\right) > 0, \qquad [A3.8]$$

which is condition [1] in the main text.

4 Altruism to same sex – marginal fitness

Consider now that the locus $\mathcal{G}$ controls altruism performed by individuals of sex i, j ∈{m,f} towards groupmates of the same sex – denoted by x_{ii}. This trait is expressed only by young adults of sex i and only impacts the competitiveness for breeding spots of individuals of sex i. Let g be the genic value of the focal young adult for this gene, G the breeding value of the focal young adult, G' the breeding value of a randomly-chosen groupmate of the focal young adult, and $\bar{G}$ the average of the population.

Assuming vanishing genetic variation – all breeding values clustered around the mean (Taylor and Frank, 1996; Frank 1997) – the direction of natural selection is given by:

$$\frac{\mathrm{d}W}{\mathrm{d}g} = \mathbb{C}_{\mathrm{i}}\frac{\mathrm{d}W_{\mathrm{i}}}{\mathrm{d}g_{\mathrm{i}}} + \mathbb{C}_{\mathrm{j}}\frac{\mathrm{d}W_{\mathrm{j}}}{\mathrm{d}g_{\mathrm{j}}}, \qquad \text{[A4.1]}$$

where $W = \mathbb{C}_{\mathrm{i}}W_{\mathrm{i}} + \mathbb{C}_{\mathrm{j}}W_{\mathrm{j}}$ is the average of relative fitness taken over sex i and sex j classes and $\mathbb{C}_{\mathrm{i}}$ and $\mathbb{C}_{\mathrm{j}}$ are the the class reproductive values for the two sexes (Fisher 1930; Taylor 1996); and with all derivatives evaluated at $g = g_{\mathrm{i}} = g_{\mathrm{j}} = \bar{G}$.

The first derivative in equation [A4.1] describes the impact of the genic value of a gene drawn from a sex-i young adult on their relative fitness. This can be expanded, employing the chain rule, obtaining:

$$\frac{\mathrm{d}W_{\mathrm{i}}}{\mathrm{d}g_{\mathrm{i}}} = \frac{\partial W_{\mathrm{i}}}{\partial x_{\mathrm{ii}}}\frac{\mathrm{d}x_{\mathrm{ii}}}{\mathrm{d}G}\frac{\mathrm{d}G}{\mathrm{d}g_{\mathrm{i}}} + \frac{\partial W_{\mathrm{i}}}{\partial x'_{\mathrm{ii}}}\frac{\mathrm{d}x'_{\mathrm{ii}}}{\mathrm{d}G'}\frac{\mathrm{d}G'}{\mathrm{d}g_{\mathrm{i}}} = \left(\frac{\partial W_{\mathrm{i}}}{\partial x_{\mathrm{ii}}}p_{\mathrm{self}} + \frac{\partial W_{\mathrm{i}}}{\partial x'_{\mathrm{ii}}}p_{\mathrm{ii}}\right)\gamma, \qquad \text{[A4.2]}$$

where $\frac{\partial W_{\mathrm{i}}}{\partial x_{\mathrm{ii}}}$ is the impact of the altruistic behaviour performed by the focal sex-i young adult on their fitness, $\frac{\partial W_{\mathrm{i}}}{\partial x'_{\mathrm{ii}}}$ is the impact of the altruistic behaviour performed by a random sex-i young adult in the focal group on the fitness of the focal sex-i young adult, $p_{\mathrm{self}} = \frac{\mathrm{d}G}{\mathrm{d}g_{\mathrm{i}}}$ is the consanguinity of the focal sex-i young adult to themselves, $p_{\mathrm{ii}} = \frac{\mathrm{d}G'}{\mathrm{d}g_{\mathrm{i}}}$ is the consanguinity

between the focal sex-i young adult and a random sex i young adult in their group, and $\gamma = \frac{\mathrm{d}x_{\mathrm{ii}}}{\mathrm{d}G} = \frac{\mathrm{d}x'_{\mathrm{ii}}}{\mathrm{d}G'}$ is the correlation between a young adult's phenotype and their breeding value.

The second derivative in equation [A4.1] describes the impact of the genic value of a gene drawn from a young adult of the other sex (sex j) on their relative fitness. Considered that the trait under consideration is only expressed by individuals of sex i and is aimed only at individuals of sex i, this derivative is null.

Altruism performed by individuals of sex i towards groupmates of sex i has an impact only on sex-i competitiveness for breeding positions t_{i} (see Methods). Therefore: $\frac{\partial W_{\mathrm{i}}}{\partial x_{\mathrm{ii}}} = \frac{\partial W_{\mathrm{i}}}{\partial t_{\mathrm{i}}}\frac{\partial t_{\mathrm{i}}}{\partial x_{\mathrm{ii}}}$, $\frac{\partial W_{\mathrm{i}}}{\partial x'_{\mathrm{ii}}} = \frac{\partial W_{\mathrm{i}}}{\partial t_{\mathrm{i}}}\frac{\partial t_{\mathrm{i}}}{\partial x'_{\mathrm{ii}}} + \frac{\partial W_{\mathrm{i}}}{\partial t'_{\mathrm{i}}}\frac{\partial t'_{\mathrm{i}}}{\partial x'_{\mathrm{ii}}}$, with $t_{\mathrm{i}} = T_{\mathrm{i}}(y,z)$ where $y = x_{\mathrm{ii}}$ and $z = x'_{\mathrm{ii}}$, and with $t'_{\mathrm{i}} = T_{\mathrm{i}}(y,z)$ where $y = x'_{\mathrm{ii}}$ and $z = x'_{\mathrm{ii}}$. We define: $\frac{\partial t_{\mathrm{i}}}{\partial y} = \frac{\partial t'_{\mathrm{i}}}{\partial y} = -\bar{t}_i\, c_{\mathrm{ii}}$ and $\frac{\partial t_{\mathrm{i}}}{\partial z} = \frac{\partial t'_{\mathrm{i}}}{\partial z} = \bar{t}_{\mathrm{i}}\, b_{\mathrm{ii}}$. Therefore we obtain: $\frac{\partial W_{\mathrm{i}}}{\partial x_{\mathrm{ii}}} = \frac{\partial W_{\mathrm{i}}}{\partial t_{\mathrm{i}}}(-\bar{t}_{\mathrm{i}}\, c_{\mathrm{ii}})$ and $\frac{\partial W_{\mathrm{i}}}{\partial x'_{\mathrm{ii}}} = \frac{\partial W_{\mathrm{i}}}{\partial t_{\mathrm{i}}}\,\bar{t}_{\mathrm{i}}\, b_{\mathrm{ii}} + \frac{\partial W_{\mathrm{i}}}{\partial t'_{\mathrm{i}}}(-\bar{t}_{\mathrm{i}}\, c_{\mathrm{ii}} + \bar{t}_{\mathrm{i}}\, b_{\mathrm{ii}})$. Substituting these expressions and equation [A4.2] into equation [A4.1] we obtain:

$$\frac{\mathrm{d}W}{\mathrm{d}g} = \mathbb{C}_{\mathrm{i}}\left(\frac{\partial W_{\mathrm{i}}}{\partial t_{\mathrm{i}}}(-c_{\mathrm{ii}}\bar{t}_{\mathrm{i}})p_{\mathrm{self}} + \left(\frac{\partial W_{\mathrm{i}}}{\partial t_{\mathrm{i}}}b_{\mathrm{ii}}\bar{t}_{\mathrm{i}} + \frac{\partial W_{\mathrm{i}}}{\partial t'_{\mathrm{i}}}(-c_{\mathrm{ii}}\bar{t}_{\mathrm{i}} + b_{\mathrm{ii}}\bar{t}_{\mathrm{i}})\right)p_{\mathrm{ii}}\right)\gamma \qquad \text{[A4.3]}$$

Population average altruism by sex i towards the same sex increases when $\frac{\mathrm{d}W}{\mathrm{d}g} > 0$. Substituting the appropriate derivatives in equation [A4.3], this condition is given by:

$$\mathbb{C}_{\mathrm{i}}(-c_{\mathrm{ii}}p_{\mathrm{self}} + (b_{\mathrm{ii}} + (\alpha_{\mathrm{i}}c_{\mathrm{ii}} - \alpha_{\mathrm{i}}b_{\mathrm{ii}}))p_{\mathrm{ii}})\gamma > 0 \qquad \text{[A4.4]}$$

If $\mathbb{C}_{\mathrm{i}} = \mathbb{C}_{\mathrm{j}} = \frac{1}{2}$ (as is the case under diploid inheritance, i.e. the case considered here; Fisher 1930; Taylor 1996), this simplifies to:

$$-c_{\mathrm{ii}}p_{\mathrm{self}} + (b_{\mathrm{ii}} + (\alpha_{\mathrm{i}}c_{\mathrm{ii}} - \alpha_{\mathrm{i}}b_{\mathrm{ii}}))p_{\mathrm{ii}} > 0 \qquad \text{[A4.5]}$$

Dividing by p_{self} to obtain relatedness coefficients ($r_{\mathrm{ii}} = \frac{p_{\mathrm{ii}}}{p_{\mathrm{self}}}$ and $r_{\mathrm{ij}} = \frac{p_{\mathrm{ij}}}{p_{\mathrm{self}}}$; see **6 Consanguinity and relatedness**) and rearranging terms, we obtain:

$$-c_{ii} + \alpha_i c_{ii} r_{ii} + (b_{ii} - \alpha_i b_{ii})\, p_{ii} > 0 \qquad [A4.6]$$

5 Altruism to opposite sex – marginal fitness

Consider now that the locus $\mathcal{G}$ controls altruism performed by individuals of sex i towards groupmates of the opposite sex, j and denoted by x_{ij} (with i, j ∈{m,f} and i ≠ j). This trait is expressed only by young adults of sex i, but impacts the competitiveness for breeding spots of individuals of both sex i and sex j in their group. Let g be the genic value of the focal young adult for this gene, G the breeding value of the focal young adult, G' the breeding value of a randomly-chosen groupmate of the focal young adult, and $\bar{G}$ the average of the population.

Assuming vanishing genetic variation – all breeding values clustered around the mean (Taylor and Frank, 1996; Frank, 1997) – the direction of natural selection is given by:

$$\frac{\mathrm{d}W}{\mathrm{d}g} = \mathbb{C}_i \frac{\mathrm{d}W_i}{\mathrm{d}g_i} + \mathbb{C}_j \frac{\mathrm{d}W_j}{\mathrm{d}g_j}, \qquad [A5.1]$$

where $W = \mathbb{C}_i W_i + \mathbb{C}_j W_j$ is the average of relative fitness taken over sex i and sex j classes and $\mathbb{C}_i$ and $\mathbb{C}_j$ are the the class reproductive values for the two sexes (Fisher 1930; Taylor 1996); and with all derivatives evaluated at $g = g_i = g_j = \bar{G}$.

The first derivative in equation [A.5.1] describes the impact of the genic value of a gene drawn from a sex-i young adult on their relative fitness. This can be expanded, employing the chain rule, obtaining:

$$\frac{\mathrm{d}W_i}{\mathrm{d}g_i} = \frac{\partial W_i}{\partial x_{ij}} \frac{\mathrm{d}x_{ij}}{\mathrm{d}G} \frac{\mathrm{d}G}{\mathrm{d}g_i} + \frac{\partial W_i}{\partial x'_{ij}} \frac{\mathrm{d}x'_{ij}}{\mathrm{d}G'} \frac{\mathrm{d}G'}{\mathrm{d}g_i} = \left(\frac{\partial W_i}{\partial x_{ij}} p_{\mathrm{self}} + \frac{\partial W_i}{\partial x'_{ij}} p_{ii}\right) \gamma, \qquad [A5.2]$$

where $\frac{\partial W_i}{\partial x_{ij}}$ is the impact of the altruistic behaviour performed by the focal sex-i young adult on their fitness, $\frac{\mathrm{d}W_i}{\mathrm{d}x'_{ij}}$ is the impact of the altruistic behaviour performed by a random sex-i young

adult in the focal group on the fitness of the focal sex-i young adult, $p_{\text{self}} = \frac{\mathrm{d}G}{\mathrm{d}g_{\text{i}}}$ is the consanguinity of the focal sex-i young adult to themselves, $p_{\text{ii}} = \frac{\mathrm{d}G'}{\mathrm{d}g_{\text{i}}}$ is the consanguinity between the focal sex-i young adult and a random sex-i young adult in their group, and $\gamma = \frac{\mathrm{d}x_{\text{ij}}}{\mathrm{d}G} = \frac{\mathrm{d}x'_{\text{ij}}}{\mathrm{d}G'}$ is the correlation between a young adult's phenotype and their breeding value.

The second derivative in equation [A5.2] describes the impact of the genic value of a gene drawn from a young adult of the other sex (sex j) on their relative fitness. Expanding it, employing the chain rule, reveals an indirect fitness component (there is no direct fitness component as sex-j young adults do not perform the behaviour). In mathematical form:

$$\frac{\mathrm{d}W_{\text{j}}}{\mathrm{d}g_{\text{j}}} = \frac{\partial W_{\text{j}}}{\partial x'_{\text{ij}}} \frac{\mathrm{d}x'_{\text{ij}}}{\mathrm{d}G'} \frac{\mathrm{d}G'}{\mathrm{d}g_{\text{j}}} = \frac{\partial W_{\text{j}}}{\partial x'_{\text{ij}}}\, p_{\text{ij}}\, \gamma\,, \qquad \text{[A5.3]}$$

where $\frac{\mathrm{d}W_{\text{j}}}{\mathrm{d}x'_{\text{ij}}}$ is the impact of the altruistic behaviour performed by a random sex-i young adult in the focal group on the fitness of the focal sex-j young adult, $p_{\text{ij}} = \frac{\mathrm{d}G'}{\mathrm{d}g_{\text{j}}}$ is the consanguinity between the focal sex-j young adult and a random sex-i young adult in their group, and $\gamma = \frac{\mathrm{d}x'_{\text{ij}}}{\mathrm{d}G'}$ is again the correlation between a young adult's phenotype and their breeding value.

Altruism performed by sex i towards sex j has an impact on both sex-i competitiveness for breeding positions t_{i} and sex-j competitiveness t_{j} (see Methods). Therefore: $\frac{\partial W_{\text{i}}}{\partial x_{\text{ij}}} = \frac{\partial W_{\text{i}}}{\partial t_{\text{i}}} \frac{\partial t_{\text{i}}}{\partial x_{\text{ij}}}$, $\frac{\partial W_{\text{i}}}{\partial x'_{\text{ij}}} = \frac{\partial W_{i}}{\partial t'_{\text{i}}} \frac{\partial t'_{\text{i}}}{\partial x'_{\text{ij}}}$, $\frac{\partial W_{\text{j}}}{\partial x'_{\text{ij}}} = \frac{\partial W_{\text{j}}}{\partial t_{\text{j}}} \frac{\partial t_{\text{j}}}{\partial x'_{\text{ij}}} + \frac{\partial W_{\text{j}}}{\partial t'_{\text{j}}} \frac{\partial t'_{\text{j}}}{\partial x'_{\text{ij}}}$, where $t_{\text{i}} = T_{\text{i}}(x_{\text{ij}})$, $t'_{\text{i}} = T_{\text{i}}(x'_{\text{ij}})$, $\bar{t}_{\text{i}} = T_{\text{i}}(\bar{x}_{\text{ij}})$, $t_{\text{j}} = t'_{\text{j}} = T_{\text{j}}(x'_{\text{ij}})$, $\bar{t}_{\text{j}} = T_{\text{j}}(\bar{x}_{\text{ij}})$ and where $\frac{\partial T_{i}(x_{\text{ij}})}{\partial x_{\text{ij}}} = \frac{\partial T_{\text{i}}(x'_{\text{ij}})}{\partial x'_{\text{ij}}} = -\,\bar{t}_{\text{i}}\, c_{\text{ij}}$ and $\frac{\partial T_{\text{j}}(x'_{\text{ij}})}{\partial x'_{\text{ij}}} = -\,\bar{t}_{\text{i}}\, b_{\text{ij}}$. Substituting these expressions and equations [A5.2] and [A5.3] into equation [A5.1] we obtain:

$$\frac{\mathrm{d}W}{\mathrm{d}g} = \mathbb{C}_{\text{i}} \left(\frac{\partial W_{\text{i}}}{\partial t_{\text{i}}} (-c_{\text{ij}}\bar{t}_{\text{i}}) p_{\text{self}} + \frac{\partial W_{\text{i}}}{\partial t'_{\text{i}}} (-c_{\text{ij}}\bar{t}_{\text{i}}) p_{\text{ii}} \right) \gamma \; + \mathbb{C}_{\text{j}} \left(\frac{\partial W_{\text{j}}}{\partial t_{\text{j}}} b_{\text{ij}}\bar{t}_{\text{j}} + \frac{\partial W_{\text{j}}}{\partial t'_{\text{j}}} b_{\text{ij}}\bar{t}_{\text{j}} \right) p_{\text{ij}}\, \gamma \qquad \text{[A5.4]}$$

Population average altruism by sex i towards sex j increases when $\frac{\mathrm{d}W}{\mathrm{d}g} > 0$. Substituting the appropriate derivatives in equation [A4.4], this condition is given by

$$\mathbb{C}_{\mathrm{i}}\left(-c_{\mathrm{ij}}p_{\mathrm{self}} + \alpha_{\mathrm{i}}c_{\mathrm{ij}}p_{\mathrm{ii}}\right)\gamma \ + \mathbb{C}_{\mathrm{j}}\left(b_{\mathrm{ij}} - \alpha_{\mathrm{i}}b_{\mathrm{ij}}\right) p_{\mathrm{ij}}\ \gamma > 0 \qquad \text{[A5.5]}$$

If $\mathbb{C}_{\mathrm{i}} = \mathbb{C}_{\mathrm{j}} = \frac{1}{2}$ (as is the case under diploid inheritance, i.e. the case considered here; Fisher 1930; Taylor 1996), this simplifies to:

$$-c_{\mathrm{ij}}p_{\mathrm{self}} + \alpha_{\mathrm{i}}c_{\mathrm{ij}}p_{\mathrm{ii}} + \left(b_{\mathrm{ij}} - \alpha_{\mathrm{i}}b_{\mathrm{ij}}\right) p_{\mathrm{ij}} > 0 \qquad \text{[A5.6]}$$

Dividing by p_{self} to obtain relatedness coefficients ($r_{\mathrm{ii}} = \frac{p_{\mathrm{ii}}}{p_{\mathrm{self}}}$ and $r_{\mathrm{ij}} = \frac{p_{\mathrm{ij}}}{p_{\mathrm{self}}}$; see **6 Consanguinity and relatedness**) and rearranging terms yields

$$-c_{\mathrm{ij}} + \alpha_{\mathrm{i}}c_{\mathrm{ij}}r_{\mathrm{ii}} + \left(b_{\mathrm{ij}} - \alpha_{\mathrm{i}}b_{\mathrm{ij}}\right) r_{\mathrm{ij}} > 0 \qquad \text{[A5.7]}$$

Notice that setting j=i in condition [A5.7] obtains condition [A4.6], i.e. the condition for increase for altruism by sex i towards other individuals of sex i. This is coincidental and is due to the two sexes having the same class reproductive values under diploidy. This allows us to present both results more compactly in one condition, i.e. condition [3] in the main text, where i, j $\in$\{m,f\} and the case i=j is allowed.

It is important to underline that this would not necessarily be possible under ploidy systems in which $\mathbb{C}_{\mathrm{i}} \neq \mathbb{C}_{\mathrm{j}}$ (e.g. haplodiploidy). Altruism towards same-sex groupmates and altruism towards opposite-sex groupmates are not the same behaviour. This can be seen by comparing conditions [A4.4] and [A5.5] (i.e. the step in the derivation immediately before the numerical values of the class reproductive values are substituted in the condition for increase).

6 Consanguinity and relatedness

The consanguinity coefficient between two individuals A and B at a given locus, p_{AB}, is defined as the probability that a gene randomly-drawn from individual A at that locus is identical-by-descent to a gene randomly-drawn from individual B at that same locus (Bulmer 1994).

The consanguinity of an individual to themselves (i.e. the case in which B=A) is given by $p_{\mathrm{self}} = \frac{1+f}{2}$. The consanguinity between adult mating partners in a post-competition group is known as 'inbreeding coefficient' and is given by $f = \varphi_{\mathrm{mf}}\, p_{\mathrm{x}}$. Similarly, the consanguinity between two sex-i adults (with i ∈{m,f}) in a post-dispersal group is given by $p_{\mathrm{ii,adult}} = \varphi_{\mathrm{ii}}\, p_{\mathrm{x}}$. The probabilities that two adults of opposite sex and two adults of sex i, who are in the same post-competition group, were born in the same group, are given by,
$\varphi_{\mathrm{mf}} = (1 - a\omega(2(1 - \sigma_{\mathrm{m}})(1 - \sigma_{\mathrm{f}}) - (1 - \sigma_{\mathrm{m}}) - (1 - \sigma_{\mathrm{f}}))(1 - m_{\mathrm{m}})(1 - m_{\mathrm{f}})$ and
$\varphi_{\mathrm{ii}} = (1 - 2a\omega\sigma_{\mathrm{i}}(1 - \sigma_{\mathrm{i}}))(1 - m_{\mathrm{i}})^2$, respectively.

The consanguinity of individuals born in the same group, p_{x}, is given by:

$$p_{\mathrm{x}} = \frac{1}{4}\left(\frac{1}{N_{\mathrm{m}}} p_{\mathrm{self}} + \frac{N_{\mathrm{m}}-1}{N_{\mathrm{m}}} p_{\mathrm{mm,adult}}\right) + \frac{1}{2} f + \frac{1}{4}\left(\frac{1}{N_{\mathrm{f}}} p_{\mathrm{self}} + \frac{N_{\mathrm{f}}-1}{N_{\mathrm{f}}} p_{\mathrm{ff,adult}}\right) \quad \text{[A6.1]}$$

Substituting in consanguinities and solving for p_{x}, we obtain:

$$p_{\mathrm{x}} = \frac{(N_{\mathrm{m}}+N_{\mathrm{f}})\, \varphi_{\mathrm{mf}}}{8\, N_{\mathrm{m}}\, N_{\mathrm{f}} - 2N_{\mathrm{f}}(N_{\mathrm{m}}-1)\, \varphi_{\mathrm{mm}} - 2N_{\mathrm{m}}(N_{\mathrm{f}}-1)\, \varphi_{\mathrm{ff}} - (4N_{\mathrm{m}}\, N_{\mathrm{f}} + N_{\mathrm{m}} + N_{\mathrm{f}})\varphi_{\mathrm{mf}}}. \quad \text{[A6.2]}$$

In a post-dispersal group, the consanguinity of a focal sex-i young adult to a random sex-i young adult is equal to the probability that the two sex-i young adults were born in the same group and that both remained in it (i.e. neither dispersed), that is $p_{\mathrm{ii}} = (1 - m_{\mathrm{i}})^2\, p_{\mathrm{x}}$. Analogously, the consanguinity of a focal sex-i young adult to a random sex-j young adult is equal to the probability that the sex-i and the sex-j young adults were born in the same group and that both remained in it (i.e. neither dispersed), that is $p_{\mathrm{ij}} = (1 - m_{\mathrm{i}})(1 - m_{\mathrm{j}})\, p_{\mathrm{x}}$.

The relatedness coefficient between two individuals A and B at a given locus, r_{AB}, is obtained by dividing p_{AB} by $p_{\mathrm{BB}} = p_{\mathrm{self}}$ (Bulmer 1994). The relatedness between two

individuals born in the same group is given by $r_{\mathrm{x}} = \frac{p_{\mathrm{x}}}{p_{\mathrm{self}}}$, the relatedness between two sex-i young adults in a post-dispersal group is given by $r_{\mathrm{ii}} = \frac{p_{\mathrm{ii}}}{p_{\mathrm{self}}} = (1 - m_{\mathrm{i}})^2\, r_{\mathrm{x}}$, and the relatedness between a sex-i young adult and a sex-j young adult in a post-dispersal group is given by $r_{\mathrm{ij}} = \frac{p_{\mathrm{ij}}}{p_{\mathrm{self}}} = (1 - m_{\mathrm{i}})(1 - m_{\mathrm{j}})\, r_{\mathrm{x}}$.

7 Demonstrations

Here, we demonstrate how sex biases in dispersal and admixture impact on pattern of sex-specific altruism. In addition, we show the effect of the total number of sex-i and sex-j adult breeders in a group on potentials for altruism.

The potential for altruism towards an individual of unspecified sex is given by $A_{\mathrm{i}} = \frac{\frac{1}{2}(r_{\mathrm{ij}} - \alpha_{\mathrm{j}} r_{\mathrm{ij}}) + \frac{1}{2}(r_{\mathrm{ii}} - \alpha_{\mathrm{i}} r_{\mathrm{ii}})}{1 - \alpha_{\mathrm{i}} r_{\mathrm{ii}}}$, the potential for altruism towards the same sex is given by $A_{\mathrm{ii}} = \frac{r_{\mathrm{ii}} - \alpha_{\mathrm{i}} r_{\mathrm{ii}}}{1 - \alpha_{\mathrm{i}} r_{\mathrm{ii}}}$, and the potential for altruism towards the opposite sex is given by $A_{\mathrm{ij}} = \frac{r_{\mathrm{ij}} - \alpha_{\mathrm{j}} r_{\mathrm{ij}}}{1 - \alpha_{\mathrm{i}} r_{\mathrm{ii}}}$, where i, j $\in$ {m,f} and i $\neq$ j.

First, we demonstrate that, assuming $M_{\mathrm{i}} = M_{\mathrm{j}}$, if $m_{\mathrm{i}} < m_{\mathrm{j}}$ then $A_{\mathrm{i}} > A_{\mathrm{j}}$; that is, all else being equal, the more philopatric sex has higher potential for altruism towards individuals of unspecified sex than the more dispersing sex. Assuming $M_{\mathrm{i}} = M_{\mathrm{j}}$, it follows that $\alpha_{\mathrm{i}} = \alpha_{\mathrm{j}} = \alpha$ and thus the potentials are $A_{\mathrm{i}} = \frac{\frac{1}{2}(r_{\mathrm{ij}} - \alpha r_{\mathrm{ij}}) + \frac{1}{2}(r_{\mathrm{ii}} - \alpha r_{\mathrm{ii}})}{1 - \alpha r_{\mathrm{ii}}}$ and $A_{\mathrm{j}} = \frac{\frac{1}{2}(r_{\mathrm{ji}} - \alpha r_{\mathrm{ji}}) + \frac{1}{2}(r_{\mathrm{jj}} - \alpha r_{\mathrm{jj}})}{1 - \alpha r_{\mathrm{jj}}}$. If $m_{\mathrm{i}} < m_{\mathrm{j}}$, then $r_{\mathrm{ii}} > r_{\mathrm{ij}} = r_{\mathrm{ji}} > r_{\mathrm{jj}}$ (see **6 Consanguinity and relatedness**) and therefore $A_{\mathrm{i}} > A_{\mathrm{j}}$.

Second, we demonstrate that, assuming $m_{\mathrm{i}} = m_{\mathrm{j}}$, if $M_{\mathrm{i}} < M_{\mathrm{j}}$ then $A_{\mathrm{i}} > A_{\mathrm{j}}$; that is, all else being equal, the sex characterised by a lower degree of admixture has higher potential for altruism towards individuals of unspecified sex than higher-admixture sex. Assuming $m_{\mathrm{i}} = m_{\mathrm{j}}$, it follows

that $r_{\mathrm{ii}} = r_{\mathrm{ij}} = r$ (see **6 Consanguinity and relatedness**) and thus the potentials are $A_{\mathrm{i}} = \frac{\frac{1}{2}(r-\alpha_{\mathrm{j}}r)+\frac{1}{2}(r-\alpha_{\mathrm{i}}r)}{1-\alpha_{\mathrm{i}}r}$ and $A_{\mathrm{j}} = \frac{\frac{1}{2}(r-\alpha_{\mathrm{i}}r)+\frac{1}{2}(r-\alpha_{\mathrm{j}}r)}{1-\alpha_{\mathrm{j}}r}$. If $M_{\mathrm{i}} < M_{\mathrm{j}}$, then $\alpha_{\mathrm{i}} > \alpha_{\mathrm{j}}$ and therefore $A_{\mathrm{i}} > A_{\mathrm{j}}$.

Third, we demonstrate that, assuming $M_{\mathrm{i}} = M_{\mathrm{j}}$, if $m_{\mathrm{i}} < m_{\mathrm{j}}$ then $A_{\mathrm{ii}} > A_{\mathrm{ij}}$; that is, all else being equal, both sexes have higher potential for altruism towards the more philopatric sex than towards the more dispersing sex. Assuming $M_{\mathrm{i}} = M_{\mathrm{j}}$, it follows that $\alpha_{\mathrm{i}} = \alpha_{\mathrm{j}} = \alpha$ and thus the potentials are $A_{\mathrm{ii}} = \frac{r_{\mathrm{ii}}-\alpha r_{\mathrm{ii}}}{1-\alpha r_{\mathrm{ii}}}$ and $A_{\mathrm{ij}} = \frac{r_{\mathrm{ij}}-\alpha r_{\mathrm{ij}}}{1-\alpha r_{\mathrm{ii}}}$. If $m_{\mathrm{i}} < m_{\mathrm{j}}$, then $r_{\mathrm{ii}} > r_{\mathrm{ij}}$ (see **6 Consanguinity and relatedness**) and therefore $A_{\mathrm{ii}} > A_{\mathrm{ij}}$.

Fourth, we demonstrate that, assuming $m_{\mathrm{i}} = m_{\mathrm{j}}$, if $M_{\mathrm{i}} > M_{\mathrm{j}}$ then $A_{\mathrm{ii}} > A_{\mathrm{ij}}$; that is, all else being equal, both sexes have higher potential for altruism towards the sex characterised by the higher degree of admixture than towards the lower-admixture sex. Assuming $m_{\mathrm{i}} = m_{\mathrm{j}}$, it follows that $r_{\mathrm{ii}} = r_{\mathrm{ij}} = r$ (see **6 Consanguinity and relatedness**) and thus the potentials are $A_{\mathrm{ii}} = \frac{r-\alpha_{\mathrm{i}}r}{1-\alpha_{\mathrm{i}}r}$ and $A_{\mathrm{ij}} = \frac{r-\alpha_{\mathrm{j}}r}{1-\alpha_{\mathrm{i}}r}$. If $M_{\mathrm{i}} > M_{\mathrm{j}}$, then $\alpha_{\mathrm{i}} < \alpha_{\mathrm{j}}$ and therefore $A_{\mathrm{ii}} > A_{\mathrm{ij}}$.

Fifth, we demonstrate that, assuming $M_{\mathrm{i}} > M_{\mathrm{j}}$ and $m_{\mathrm{i}} < m_{\mathrm{j}}$, then it is always true that $A_{\mathrm{ii}} > A_{\mathrm{ij}}$; that is, both sexes have higher potential for altruism towards the sex that is characterised by the higher degree of admixture and is more philopatric, than towards the opposite sex. Assuming $M_{\mathrm{i}} > M_{\mathrm{j}}$, it follows that $\alpha_{\mathrm{i}} < \alpha_{\mathrm{j}}$ and assuming $m_{\mathrm{i}} < m_{\mathrm{j}}$ it follows that $r_{\mathrm{ii}} > r_{\mathrm{ij}}$. Therefore $(1 - \alpha_{\mathrm{i}})r_{\mathrm{ii}} > (1 - \alpha_{\mathrm{j}})r_{\mathrm{ij}}$, which implies $A_{\mathrm{ii}} > A_{\mathrm{ij}}$.

Sixth, we demonstrate that, assuming $M_{\mathrm{i}} > M_{\mathrm{j}}$ and $m_{\mathrm{i}} > m_{\mathrm{j}}$, then it follows that $A_{\mathrm{ii}} > A_{\mathrm{ij}}$ or $A_{\mathrm{ii}} < A_{\mathrm{ij}}$ depending on values of M_{i}, M_{j}, m_{i}, m_{j}; that is, both sexes may have higher potential for altruism towards the sex that is characterised by higher admixture and a higher rate of dispersal than towards the opposite sex, depending on parameter values. Assuming $M_{\mathrm{i}} > M_{\mathrm{j}}$, it follows that $\alpha_{\mathrm{i}} < \alpha_{\mathrm{j}}$ and assuming $m_{\mathrm{i}} > m_{\mathrm{j}}$ it follows that $r_{\mathrm{ii}} < r_{\mathrm{ij}}$. Therefore it may be that $(1 - \alpha_{\mathrm{i}})r_{\mathrm{ii}} > (1 - \alpha_{\mathrm{j}})r_{\mathrm{ij}}$ (which implies $A_{\mathrm{ii}} > A_{\mathrm{ij}}$) or it may be that $(1 - \alpha_{\mathrm{i}})r_{\mathrm{ii}} < (1 - \alpha_{\mathrm{j}})r_{\mathrm{ij}}$ (which implies $A_{\mathrm{ii}} < A_{\mathrm{ij}}$).

Seventh, we demonstrate that A_i is a decreasing function of N_i and N_j; that is the higher the numbers of sex-i and sex-j breeders in a group, the lower the potential for altruism by sex i. The potential is $A_\mathrm{i} = \frac{\frac{1}{2}(r_\mathrm{ij} - \alpha_\mathrm{j} r_\mathrm{ij}) + \frac{1}{2}(r_\mathrm{ii} - \alpha_\mathrm{i} r_\mathrm{ii})}{1 - \alpha_\mathrm{i} r_\mathrm{ii}}$, where $\alpha_\mathrm{i} = 1 - 2a\omega M_\mathrm{i}$, $r_\mathrm{ii} = (1 - m_\mathrm{i})^2 r_\mathrm{x}$ and $r_\mathrm{ij} = (1 - m_\mathrm{i})(1 - m_\mathrm{j}) r_\mathrm{x}$. Considered that $\frac{\mathrm{d}A_\mathrm{i}}{\mathrm{d}r_\mathrm{x}} > 0$, and $\frac{\mathrm{d}r_\mathrm{x}}{\mathrm{d}p_\mathrm{x}} > 0$, and $\frac{\mathrm{d}p_\mathrm{x}}{\mathrm{d}N_\mathrm{i}}, \frac{\mathrm{d}p_\mathrm{x}}{\mathrm{d}N_\mathrm{j}} < 0$ (**6 Consanguinity and relatedness**), it follows that $\frac{\mathrm{d}A_\mathrm{i}}{\mathrm{d}N_\mathrm{i}}, \frac{\mathrm{d}A_\mathrm{i}}{\mathrm{d}N_\mathrm{i}} < 0$.

Eighth, we demonstrate that A_ii and A_ij is a decreasing function of N_i and N_j; that is the higher the numbers of sex-i and sex-j breeders in a group, the lower the potential for altruism by sex i towards sex-i groupmates. The potentials are $A_\mathrm{ii} = \frac{r_\mathrm{ii} - \alpha_\mathrm{i} r_\mathrm{ii}}{1 - \alpha_\mathrm{i} r_\mathrm{ii}}$ and $A_\mathrm{ij} = \frac{r_\mathrm{ij} - \alpha_\mathrm{j} r_\mathrm{ij}}{1 - \alpha_\mathrm{i} r_\mathrm{ii}}$, where $\alpha_\mathrm{i} = 1 - 2a\omega M_\mathrm{i}$, $r_\mathrm{ii} = (1 - m_\mathrm{i})^2 r_\mathrm{x}$ and $r_\mathrm{ij} = (1 - m_\mathrm{i})(1 - m_\mathrm{j}) r_\mathrm{x}$. Considered that $\frac{\mathrm{d}A_\mathrm{ii}}{\mathrm{d}r_\mathrm{x}}, \frac{\mathrm{d}A_\mathrm{ij}}{\mathrm{d}r_\mathrm{x}} > 0$, and $\frac{\mathrm{d}r_\mathrm{x}}{\mathrm{d}p_\mathrm{x}} > 0$, and $\frac{\mathrm{d}p_\mathrm{x}}{\mathrm{d}N_\mathrm{i}}, \frac{\mathrm{d}p_\mathrm{x}}{\mathrm{d}N_\mathrm{j}} < 0$ (**6 Consanguinity and relatedness**), it follows that $\frac{\mathrm{d}A_\mathrm{ii}}{\mathrm{d}N_\mathrm{i}}, \frac{\mathrm{d}A_\mathrm{ii}}{\mathrm{d}N_\mathrm{j}}, \frac{\mathrm{d}A_\mathrm{ij}}{\mathrm{d}N_\mathrm{i}}, \frac{\mathrm{d}A_\mathrm{ij}}{\mathrm{d}N_\mathrm{j}} < 0$.

Ninth, we demonstrate that, independently of the values of N_i and N_j, $A_\mathrm{i}/A_\mathrm{j}$ is a constant; that is the numbers of sex-i and sex-j adult breeders in a group do not influence the potential for altruism of sex-i relative to that of sex-j. The potentials are $A_\mathrm{i} = \frac{\frac{1}{2}(r_\mathrm{ij} - \alpha_\mathrm{j} r_\mathrm{ij}) + \frac{1}{2}(r_\mathrm{ii} - \alpha_\mathrm{i} r_\mathrm{ii})}{1 - \alpha_\mathrm{i} r_\mathrm{ii}}$ and $A_\mathrm{j} = \frac{\frac{1}{2}(r_\mathrm{ji} - \alpha_\mathrm{i} r_\mathrm{ji}) + \frac{1}{2}(r_\mathrm{jj} - \alpha_\mathrm{j} r_\mathrm{jj})}{1 - \alpha_\mathrm{j} r_\mathrm{jj}}$, where $\alpha_\mathrm{i} = 1 - 2a\omega M_\mathrm{i}$, $r_\mathrm{ii} = (1 - m_\mathrm{i})^2 r_x$ and $r_\mathrm{ij} = (1 - m_\mathrm{i})(1 - m_\mathrm{j}) r_x$ (see **6 Consanguinity and relatedness**). Thus, $A_\mathrm{i}/A_\mathrm{j} = \frac{\frac{1}{2}(r_\mathrm{ij} - \alpha_\mathrm{j} r_\mathrm{ij}) + \frac{1}{2}(r_\mathrm{ii} - \alpha_\mathrm{i} r_\mathrm{ii})}{\frac{1}{2}(r_\mathrm{ji} - \alpha_\mathrm{i} r_\mathrm{ji}) + \frac{1}{2}(r_\mathrm{jj} - \alpha_\mathrm{j} r_\mathrm{jj})} =$
$\frac{\frac{1}{2}(1 - \alpha_\mathrm{j})(1 - m_\mathrm{i})(1 - m_\mathrm{j}) r_x + \frac{1}{2}(1 - \alpha_\mathrm{i})(1 - m_\mathrm{i})^2 r_x}{\frac{1}{2}(1 - \alpha_\mathrm{i})(1 - m_\mathrm{i})(1 - m_\mathrm{j}) r_x + \frac{1}{2}(1 - \alpha_\mathrm{j})(1 - m_\mathrm{j})^2 r_x} = \frac{(1 - \alpha_\mathrm{j})(1 - m_\mathrm{i})(1 - m_\mathrm{j}) + (1 - \alpha_\mathrm{i})(1 - m_\mathrm{i})^2}{(1 - \alpha_\mathrm{i})(1 - m_\mathrm{i})(1 - m_\mathrm{j}) + (1 - \alpha_\mathrm{j})(1 - m_\mathrm{j})^2}$.

Tenth, we demonstrate that, independently of the values of N_i and N_j, $A_\mathrm{ii}/A_\mathrm{ij}$ is a constant; that is the numbers of sex-i and sex-j adult breeders in a group do not influence the potential for altruism of sex-i towards sex-i groupmates relative to the potential for altruism of sex-i towards

sex-j groupmates. The potentials are $A_{\text{ii}} = \frac{r_{\text{ii}} - \alpha_{\text{i}} r_{\text{ii}}}{1 - \alpha_{\text{i}} r_{\text{ii}}}$ and $A_{\text{ij}} = \frac{r_{\text{ij}} - \alpha_{\text{j}} r_{\text{ij}}}{1 - \alpha_{\text{i}} r_{\text{ii}}}$, where $\alpha_{\text{i}} = 1 - 2a\omega M_{\text{i}}$, $r_{\text{ii}} = (1 - m_{\text{i}})^2 r_x$ and $r_{\text{ij}} = (1 - m_{\text{i}})(1 - m_{\text{j}}) r_x$ (see **6 Consanguinity and relatedness**).. Thus, $A_{\text{ii}}/A_{\text{ij}} = \frac{r_{\text{ii}} - \alpha_{\text{i}} r_{\text{ii}}}{r_{\text{ij}} - \alpha_{\text{j}} r_{\text{ij}}} = \frac{(1-\alpha_{\text{i}})(1-m_{\text{i}})^2 r_x}{(1-\alpha_{\text{j}})(1-m_{\text{i}})(1-m_{\text{j}}) r_x} = \frac{(1-\alpha_{\text{i}})(1-m_{\text{i}})^2}{(1-\alpha_{\text{j}})(1-m_{\text{i}})(1-m_{\text{j}})}$.

Notice that, assuming $M_{\text{i}} = M_{\text{j}}$ and $m_{\text{i}} = m_{\text{j}}$, it follows that $A_{\text{i}} = A_{\text{j}} = A_{\text{ii}} = A_{\text{ij}}$ for any value of N_{i} and N_{j}.

8 Additional references

Bulmer, M., 1994. *Theoretical Evolutionary Ecology.* Sunderland, MA: Sinauer Associates.

Fisher, R. A., 1930. *The Genetical Theory of Natural Selection.* Oxford, UK: The Clarendon Press.

Price, G. R. and Smith, C. A. B., 1972. Fisher's Malthusian parameter and reproductive value. *Ann Hum. Genet.,* 36, pp.1-7.
